# Synthesis and biological investigations of 3β-aminotropane arylamide derivatives with atypical antipsychotic profile

**DOI:** 10.1007/s00044-018-2203-z

**Published:** 2018-06-22

**Authors:** Jacek Stefanowicz, Tomasz Słowiński, Martyna Z. Wróbel, Grzegorz Ślifirski, Maciej Dawidowski, Zdzisława Stefanowicz, Magdalena Jastrzębska-Więsek, Anna Partyka, Anna Wesołowska, Jadwiga Turło

**Affiliations:** 1 Department of Drug Technology and Pharmaceutical Biotechnology, Faculty of Pharmacy, Medical University of Warsaw, 1 Banacha Street, 02-097 Warsaw, Poland; 2 Department of Inorganic and Analytical Chemistry, Faculty of Pharmacy, Medical University of Warsaw, 1 Banacha Street, 02-097 Warsaw, Poland; 3 Department of Clinical Pharmacy, Jagiellonian University Medical College, 9 Medyczna Street, 30-688 Cracow, Poland

**Keywords:** Atypical antipsychotics, 3β-aminotropane derivatives, 5-HT_1A_, 5-HT_2A_, D_2_, Dopamine receptor ligands

## Abstract

This work is a continuation of our previous research, concentrating this time on lead structure modification to increase the 5-HT_1A_ receptor affinity and water solubility of designed compounds. Therefore, the compounds synthesised within the present project included structural analogues of 3β-acylamine derivatives of tropane with the introduction of a methyl substituent in the benzyl ring and a 2-quinoline, 3-quinoline, or 6-quinoline moiety. A series of novel 3β**-**aminotropane derivatives was evaluated for their affinity for 5-HT_1A_, 5-HT_2A_, and D_2_ receptors, which allowed for the identification of compounds **12e**, **12i**, and **19a** as ligands with highest affinity for the tested receptors; they were then subjected to further evaluation in preliminary in vivo studies. Selected compounds **12i** and **19a** displayed antipsychotic properties in the d-amphetamine-induced and MK-801-induced hyperlocomotor activity test in mice. Moreover, compound **19a** showed significant antidepressant-like activity in the forced swim test in mice.

## Introduction

The drug files for typical and atypical, including the latest, antipsychotics (aripiprazole, brexpiprazole, cariprazine) on FDA’s Accessdata drug data base website contain the information that their mechanism of action is unknown (FDA 2008a, 2013b, 2014c, 2014d, 2015e, 2015f, 2015g, 2015h). However, in vitro studies show that all antipsychotic drugs bind to D_2_ receptors and the dosage correlates with the strength of affinity for these receptors. Dosage has not been shown to correlate with affinity for non-D_2_ receptors (Rzewuska 2009). First-generation, or typical, antipsychotics, such as chlorpromazine or haloperidol are antagonists of D_2_ receptors, while second-generation, or atypical, antipsychotics (clozapine, olanzapine, risperidone) are described as antagonists of 5-HT_2A_/D_2_, the antagonistic effect on 5-HT_2A_ receptors being greater than that against D_2_ receptors (Meltzer 2013; Möller et al. 2015). The three newest antipsychotic drugs listed above are characterised as being partial agonists of D_2_ receptors (Citrome 2015; Stahl 2015, 2016; Frankel and Schwartz 2017).

These findings indicate that inhibition of dopaminergic transmission appears to be fundamentally important in the treatment of symptoms of schizophrenia.

The dopaminergic hypothesis also furnishes the best known explanation of the relation between neurochemical factors and the clinical manifestations of schizophrenia. Historically, the hypothesis was made increasingly more precise in three stages (Howes and Kapur 2009). The first concept (I), finally formulated in the 1970’s, was called the dopamine receptor hypothesis. The main focus was on dopaminergic hyperactivity, the control of which via blocking dopamine receptors was supposed to provide effective therapy (Creese et al. 1976). Thus, it was a very general idea that did not account for differences between individual dimensions of schizophrenia (e.g., classification of symptoms), risk factors or the relation between different levels of dopamine noted in various areas within the CNS and the clinical expression of symptoms (Snyder 1976). The second concept (II) of 1991 already considered differences in dopaminergic activity between individual regions of the cerebrum, also with regard to various subtypes of the receptor (D_1_ vs. D_2_) (Davis et al. 1991). The main assumption of that concept was frontal dopaminergic hypoactivity resulting in striatal dopaminergic hyperactivity. The negative symptoms of schizophrenia were explained in terms of inadequate dopaminergic transmission in the frontal cortical areas and the positive symptoms were attributed to increased dopaminergic transmission in subcortical nuclei. This concept also had its weaknesses such as the absence of direct evidence at that time for low cortical dopamine levels, ignoring the fact that cortical phenomena are more complex than “hypofrontality” alone, lack of a model linking those abnormalities with clinical phenomena (e.g., the link between dopaminergic hyperactivity and delusions) or failure to include the aetiology of dopaminergic imbalance in schizophrenia (Davis et al. 1991; Tsoi et al. 2008; Howes and Kapur 2009). The third concept (III) (2009) is based on four core assumptions:

- risk factors for schizophrenia are linked with one another and result in dysregulation of dopaminergic neurotransmission. Regardless of the cause, it is this dysregulation that is the starting point for the development of psychosis in schizophrenia;
- dysregulation of dopaminergic transmission occurs at the level of presynaptic control rather than, as was previously believed, postsynaptic D_2_ receptors;
- dopaminergic dysregulation may be associated with psychosis rather than schizophrenia, and, for most of its duration, with susceptibility to psychosis. An individual’s diagnosis may be related to the type of factors that have produced the psychosis and to socio-cultural conditions;
- dopamine dysregulation may alter an individual’s assessment of stimuli, perhaps as a result of impaired salience (Howes and Kapur 2009).

The first assumption refers to factors associated with the prenatal period and early childhood, the environment and genetic determinants. It assumes that these are the factors that dopaminergic dysfunction, an abnormality consisting in increased striatal levels of dopamine, is associated with (Haleem 2015; Howes et al. 2017). This assumption changes the approach to antipsychotic therapy and is based on observations indicating that currently available antipsychotics do not treat the underlying abnormalities. Antipsychotics influences the postsynaptic effects of abnormal dopamine release, while the actual problem occurs at an earlier (presynaptic) stage (second assumption). The third and fourth assumptions address psychosis as a salience syndrome. Dopaminergic dysregulation in schizophrenia is viewed here as an extremely significant, but not the only, component contributing to onset of clinical symptoms. Changes in many neuronal and neurotransmitter systems, combined with other biological and environmental factors, lead to dopaminergic hyperactivity in the striatum. It can be said that dopaminergic dysregulation that has reached a certain level of severity, combined with corresponding clinical phenomena, such as delusions and hallucinations, leads to a diagnosis of psychosis and/or schizophrenia.

Other neurotransmitters should also be considered besides dopamine (Howes and Kapur 2009). In this regard, significant interaction is hypothesised to take place between dopaminergic and serotonergic pathways. Influence on serotonergic receptors may be the underlying cause of cognitive disturbances and negative symptoms seen in psychoses and mood disorders (Meltzer and Massey 2011; Sumiyoshi et al. 2014). Clozapine, the pioneering atypical antipsychotic with known efficacy against negative symptoms, acts as a 5-HT_2A_ receptor antagonist (Meltzer and Massey 2011). It demonstrates a much lower affinity for the D_2_ receptor compared to classical neuroleptics. Similar properties are exhibited by other atypical antipsychotics, namely olanzapine, risperidone, zotepine, sertindole, quetiapine or ziprasidone, whose discovery was greatly influenced by Meltzer et al.’s hypothesis that compounds of this class should be characterised by a particular 5-HT_2A_/D_2_ p*K*_i_ ratio (Meltzer’s Index) (Meltzer et al. 1989; Meltzer and Huang 2008; Meltzer and Massey 2011). Cortical 5-HT_2A_ receptors may play a key role in the development of psychoses via modulating intracortical and cortico-subcortical glutamatergic transmission (Meltzer and Huang 2008). Several new antipsychotic and antidepressant medications (cariprazine, brexpiprazole, quetiapine) reduce the severity of negative symptoms also partly via 5-HT_1A_ receptors, while producing milder extrapyramidal symptoms (Newman-Tancredi and Albert 2012; Sumiyoshi et al. 2014; Haleem 2015). Taking advantage of action at this receptor may positively influence patients’ motivation (Neves et al. 2010).

**Fig. 1:**
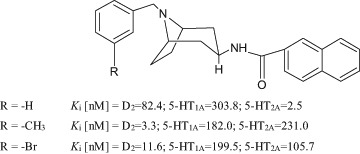
Arylamide derivatives of 3β-aminotropane

Our previous publications have described the synthesis and biological activity studies of new derivatives of 3β-aminotropane (Słowiński et al. 2011, 2013; Stefanowicz et al. 2016). Some of these derivatives demonstrate high activity at the D_2_, 5HT_1A_, and 5-HT_2A_ receptors (Fig. 1).

The binding affinity profiles of these structures are similar to those of the recognised atypical antipsychotics clozapine (*K*_i_ [nM]=D_2_ = 130; 5-HT_1A_ = 140; 5-HT_2A_ = 8.9) and quetiapine (*K*_i_ [nM] = D_2_ = 180; 5-HT_1A_ = 230; 5-HT_2A_ = 220) (Schmidt et al. 2001).

**Fig. 2:**
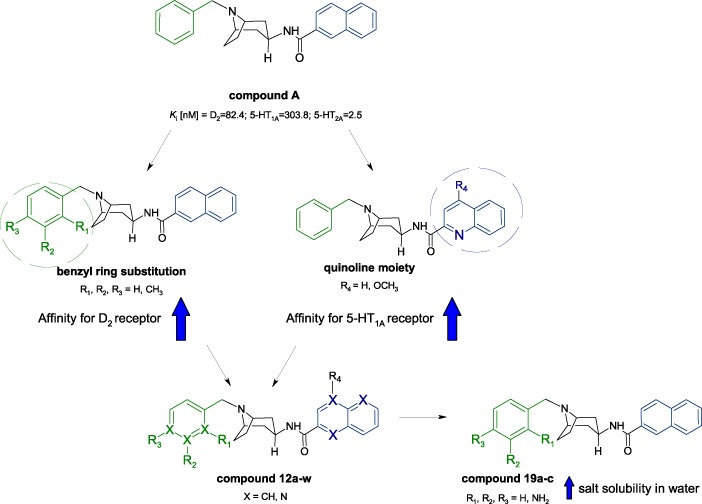
Rationale of the planned structural modifications

The aim of our work is to continue the search for new compounds with antipsychotic potential in this group of derivatives. We decided to synthesise and analyse analogues of the above structures containing an additional nitrogen atom in the molecule (Fig. 2). The addition of a nitrogen atom will certainly influence their biological activity and will make it possible to study electron effects on binding affinity for selected molecular targets, while also improving water solubility of salts of these new compounds as salts of the exemplary structures (Fig. 1) are characterised by very poor solubility.

The additional nitrogen atom is placed in a naphthalene system, producing quinoline and isoquinoline derivatives, or/and in a phenyl ring, producing pyridine derivatives, or in the form of an amine moiety, as a substituent of the phenyl ring (Fig. 2). The linking position for the quinoline and isoquinoline systems remains the same as in 2-naphthalene derivatives as this configuration generates much better affinity for the D_2_, 5-HT_1A_, and 5-HT_2A_ receptors than in 1-naphthalene analogues (Słowiński et al. 2011; Zajdel et al. 2012). We are proceeding with synthesis of equatorial isomers (β) only as they possess much better affinity for the receptors of interest than their axial (α) analogues (Słowiński et al. 2011; Stefanowicz et al. 2016).

## Material and methods

### Chemistry

#### General remarks

**Scheme 1:**
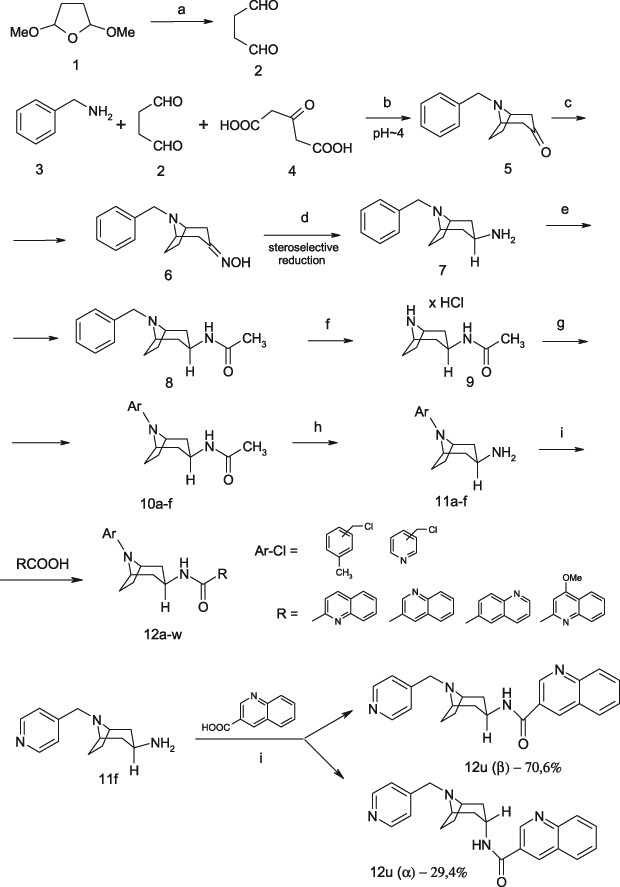
Reagents: **a** dil. HCl; **b** NaOH, HCl. q.s.; **c** NH_2_OH × HCl; NaHCO_3_, EtOH; **d** BuOH/Na, **e** CHCl_3_, CH_3_COCl; **f** H_2_, 10%Pd/C, EtOH/HCl; **g** ArCl, acetone, K_2_CO_3_ anh., KI; **h** 10%H_2_SO_4_; **i** RCOOH, ClCOOEt, TEA, DMF

**Scheme 2:**
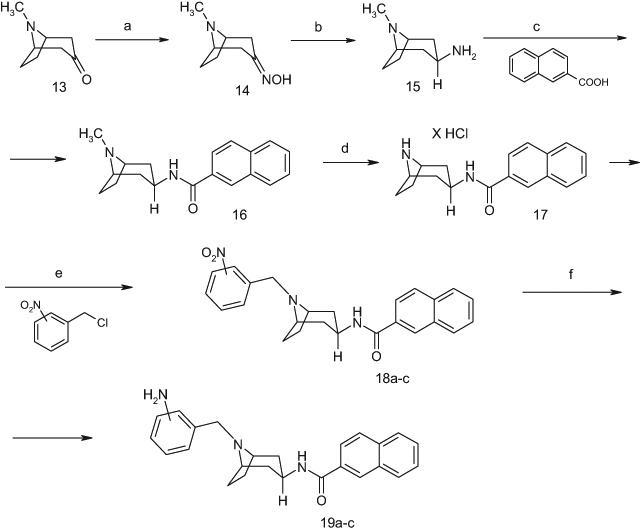
Reagents: **a** NH_2_OH x HCl; NaHCO_3_, EtOH; **b** Na/PrOH; **c** ClCOOEt, TEA, DMF, 2-naphthoic acid; **d** /I/ ClCOOCHClCH_3_, DMC, ClCH_2_CH_2_Cl, /II/ MeOH; **e** acetone, K_2_CO_3_, anh. KI, o-nitrobenzylchlorides, m-nitrobenzylchlorides, p-nitrobenzylchlorides; **f** H_2_, PtO_2_, EtOH

**Fig. 3:**
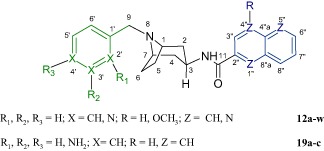
Numbering system for NMR spectra interpretation

All solvents and raw materials were purchased from commercial sources. Column chromatography was carried out using a Merck Silica gel 60 A (63–200 µm) column as the stationary phase and chloroform:methanol (9:1 v/v) as eluent. Melting points were determined on an Electrothermal 9100 apparatus with open capillary tubes and were uncorrected. IR spectra were obtained using a Shimadzu FTIR-8300 spectrometer. NMR spectra were recorded on a Varian Inova 500 (500 MHz) spectrometer. Chemical shifts (*δ*) were expressed in parts per million (ppm) relative to tetramethylsilane used as the internal reference. The following abbreviations are used to describe peak patterns when appropriate: s (singlet), bs (broad singlet), d (doublet), dd (double doublet), t (triplet), td (triple doublet), pt (pseudo triplet), 4d (quartet of doublets), m (multiplet), q (quartet), qu (quintet), * (overlapping signals). Coupling constants (*J*) are in hertz (Hz). ESI-HRMS spectra were obtained on an LCT TOF (Micromass) instrument. Intermediate **8** and **9** (Scheme 1) was obtained following the protocol in Ref. (Słowiński et al. 2011; Dostert et al. 1984). Intermediate **16** and **17** (Scheme 2) was obtained following the protocol in Ref. (Stefanowicz et al. 2016; Dostert et al. 1984) (see Supplementary material). The ^1^H NMR spectra of all considered final compounds are presented in Supplementary material. The purity of the tested compounds was determined and was higher than 95% (Fig. 3).

#### General procedure for synthesis of acetamides (**10a**–**f**)

The 0.02 mol of 8-azabicyclo[3.2.1]oct-3β-ylacetamide hydrochloride (**9**), 0.02 mol of appropriate arylmethyl chloride (2-methylbenzyl chloride for **10a**, 3-methylbenzyl chloride for **10b** or 4-methylbenzyl chloride for **10c**, 2-(chloromethyl)pyridine hydrochloride for **10d**, 3-(chloromethyl)pyridine hydrochloride for **10e**, 4-(chloromethyl)pyridine hydrochloride for **10f**), 0.09 mol anhydrous K_2_CO_3_ and catalytic amount of KI were suspended in 80 mL of acetone. The reaction mixture was refluxed with stirring for 2–3 h under TLC control. The solution was cooled and solvent was removed in vacuo, residue was dissolved in mixture of 40 mL water and 40 mL chloroform (exception is compound **10e**). The solution was extracted with chloroform (2 × 40 mL). The combined organic extracts were dried with magnesium sulphate, filtered, and the solvent was evaporated in vacuo. The solid residue was purified by crystallisation.

##### N-[8-(2-methylbenzyl)-8-azabicyclo[3.2.1]oct-3β-yl]acetamide (**10a**)

Crystallisation from ethyl acetate: ethanol 4:1. Yield: 4.5 g (70.2%); m.p. 198.6–199.8 °C; ^1^H NMR (500 MHz, CDCl_3_): *δ* m 7.31 (C3’H); m 7.14 (C4’H, C5’H, C6’H); d 5.53 (NH), ^3^*J* = 7.5; m 4.11 (C3H), ^3^*J*_A–A_ = 11.5, ^3^*J*_A–E_ = 7.0*; s 3.45 (C9H_2_); pt 3.18 (C1H, C5H); s 2.36 (C10H_3_); m 2.03 (C2H(E), C4H(E)); s 1.92 (C13H_3_); m 1.79 (C6H(E), C7H(E)); pk 1.71 (C6H(A), C7H(A)); pt 1.47 (C2H(A), C4H(A)) *-the signal of the C3H proton it has the form of multiplet. It is formed of 3 overlapping quartets, calculating the constants of coupling it can be assume that the C3H proton is axial; ^13^C NMR (125 MHz, CDCl_3_): *δ* 169.2 (C12); 138.0 (C2’); 137.0 (C1’); 130.1 (C3’); 128.7 (C6’); 126.6 (C4’); 125.4 (C5’); 58.9 (C1, C5); 54.4 (C9); 41.5 (C3); 38.8 (C2, C4); 26.4 (C6, C7); 23.4 (C13); 19.1 (C10); IR (KBr) cm^−1^: ν3258 (NH), 1650 (CO); ESI-HRMS *m*/*z* calcd for C_17_H_24_N_2_OH (M + H)^+^ 273.1967, found 273.1975.

##### N-[8-(3-methylbenzyl)-8-azabicyclo[3.2.1]oct-3β-yl]acetamide (**10b**)

Crystallisation from ethyl acetate: hexane 3:1. Yield: 2.1 g (46.42%) synthesised from 0.016 mol **9**; m.p. 135.2–136.2 °C; ^1^H NMR (500 MHz, CDCl_3_): *δ* t 7.20 (C5’H), ^3^*J* = 7.5; m 7.17 (C2’H, C6’H); d 7.06 (C4’H), ^3^*J* = 7.0; d 5.22 (NH), ^3^*J* = 7.0; m 4.14 (C3H(axial)); s 3.50 (C9H_2_); pt 3.22 (C1H, C5H); s 2.34 (C10H_3_); m 2.03 (C2H(E), C4H(E)); s 1.93 (C13H_3_); m 1.81 (C6H(E), C7H(E)); m 1.72 (C6H(A), C7H(A)); td 1.51 (C2H(A), C4H(A)), ^3^*J*_A-A_ = 12.5, ^3^*J*_A-E_ = 2.0; ^13^C NMR (125 MHz, CDCl_3_): *δ* 169.2 (C12); 139.7 (C1’); 137.8 (C3’); 129.4 (C6’); 128.1 (C5’); 127.6 (C2’); 125.7 (C4’); 58.8 (C1, C5); 56.3 (C9); 41.5 (C3); 38.6 (C2, C4); 26.4 (C6, C7); 23.6 (C13); 21.4 (C10); IR (KBr) cm^−1^: ν3265 (NH), 1624 (CO); ESI-HRMS *m*/*z* calcd for C_17_H_24_N_2_OH (M + H)^+^ 273.1967, found: 273.1966.

##### N-[8-(4-methylbenzyl)-8-azabicyclo[3.2.1]oct-3β-yl]acetamide (**10c**)

Crystallisation from ethyl acetate. Yield: 0.86 g (63.0%) synthesised from 0.005 mol **9**; m.p. 169.1–171.7 °C; ^1^H NMR (CDCl_3_, 500 MHz): *δ* d 7.24 (C3’H, C5’H), ^3^*J* = 8.5; d 7.12 (C2’H, C6’H), ^3^*J* = 7.5; d5.31 (NH), ^3^*J* = 7.5; m 4.13 (C3H(axial)), s 3.48 (C9H_2_); pt 3.19 (C1H, C5H); s 2.33 (C10H_3_); m 2.02 (C6H(E), C7H(E); s 1.93 (C12H_3_); m 1.79 (C2H(E), C4H(E); m 1.70 (C6H(A), C7H(A); td 1.48 (C2H(A), C4H(A), ^3^*J*_A–A_ = 12.5, ^3^*J*_A–E_ = 2.5); ^13^C NMR (CDCl_3_, 125 MHz): *δ* 169.2 (C11); 136.9 (C1’); 136.3 (C4’); 128.9 (C2’, C6’); 128.5 (C3’, C5’); 58.7 (C1, C5); 56.0 (C9); 41.5 (C3); 38.6 (C2, C4); 26.4 (C6, C70; 23.6 (C12); 21.1 (C10); IR (KBr) cm^−1^: ν3268 (NH), 1634 (CO); ESI-HRMS *m*/*z* calcd for C_17_H_24_N_2_OH (M + H)^+^ 273.1967, found: 273.1957.

##### N-[8-(2-pyridylmethyl)-8-azabicyclo[3.2.1]oct-3β-yl]acetamide (**10d**)

Crystallisation from acetone. Yield: 3.31 g (74.72%) synthesised from 0.017 mol **9**; m.p. 170.2–171.5 °C; ^1^H NMR (500 MHz, CDCl_3_): *δ* 4d 8.52 (C3’H), ^3^*J* = 5.0, ^4^*J* = 2.0, ^5^*J* = 1.0; td 7.66 (C5’H), ^3^*J* = 8.0, ^4^*J* = 1.5; d 7.53 (C6’H), ^3^*J* = 7.5; 4d 7.15 (C4’H), ^3^*J*_1_ = 7.5, ^3^*J*_2_ = 5.0, ^4^*J* = 1.5; d 5.34 (NH), ^3^*J* = 7.7; m 4.16 (C3H(axial)); s 3.70 (C9H_2_); pt 3.24 (C1H, C5H); m 2.07 (C2H(E), C4H(E)); s 1.94 (C12H_3_); m 1.82 (C6H(E), C7H(E)); m 1.74 (C6H(A), C7H(A)); td 1.56 (C2H(A), C4H(A)), ^3^*J*_A-A_ = 12.5, ^3^*J*_A–E_ = 2.5; ^13^C NMR (125 MHz, CDCl_3_): *δ* 169.2 (C11); 160.2 (C1’); 149.0 (C3’); 136.4 (C5’); 122.5 (C6’); 121.8 (C4’); 59.1 (C1, C5); 58.1 (C9); 41.3 (C3); 38.1 (C2, C4); 26.6 (C6, C7); 23.5 (C12); IR (KBr) cm^−1^: ν3250 (NH), 1639 (CO); ESI-HRMS *m*/*z* calcd for C_15_H_21_N_3_ONa (M + Na)^+^ 282.1582, found: 282.1581.

##### N-[8-(3-pyridylmethyl)-8-azabicyclo[3.2.1]oct-3β-yl]acetamide (**10e**)

Synthesised from 0.009 mol **9**. Reaction was carried out under nitrogen atmosphere for 2.5 h. The solution was cooled to 0 °C and the 50 mL of 10% H_2_SO_4_ was added dropwise with constant stirring. Inorganic precipitate was filtered and the acetone was evaporated in vacuo. The crude compound **10e** (as a solution in 10% H_2_SO_4_) was used in the next step of synthesis without further purification because its instability.

##### N-[8-(4-pyridylmethyl)-8-azabicyclo[3.2.1]oct-3β-yl]acetamide (**10f**)

Crystallisation from acetone. Yield: 3.15 g (58.4%) synthesised from 0.016 mol **9**; m.p. 187.5–190.5 °C; ^1^H NMR (500 MHz, CDCl_3_): *δ* dd 8.53 (C2’H, C6’H), ^3^*J* = 4.5, ^4^*J* = 1.5; d 7.32 (C3’H, C5’H), ^3^*J* = 4.5; d 5.62 (NH), ^3^*J* = 7.5; m 4.15 (C3H(axial)); s 3.53 (C9H_2_); pt 3.17 (C1H, C5H); m 2.02 (C2H(E), C4H(E)); s 1.95 (C12H_3_); m 1.84 (C6H(E), C7H(E)); pk 1.75 (C6H(A), C7H(A)); td 1.53 (C2H(A), C4H(A)), ^3^*J*_A–A_ = 12.5, ^3^*J*_A–E_ = 2.5; ^13^C NMR (125 MHz, CDCl_3_): *δ* 169.3 (C11); 149.6 (C2’, C6’); 149.4 (C4’); 123.3 (C3’, C5’); 59.2 (C1, C5); 55.4 (C9); 41.2 (C3); 38.5 (C2, C4); 26.4 (C6, C7); 23.5 (C12); IR (KBr) cm^−1^: ν3253 (NH), 1632 (CO); ESI-HRMS *m*/*z* calcd for C_15_H_21_N_3_ONa (M + Na)^+^ 282.1582, found: 282.1594.

#### General procedure for synthesis of amines (**11a**–**f**)

A solution of 0.02 mol appropriate acetamide derivative (**10a–f**) and 68 mL 10% H_2_SO_4_ was refluxed with stirring. The reaction time was determined using TLC. The reaction mixture was cooled to room temperature and then alkalised with saturated aqueous solution of NaOH (to a pH of 10–12), diluted with 80 mL of mixture H_2_O and CH_2_Cl_2_ 1:1, next the aqueous phase was extracted with CH_2_Cl_2_ (3 × 40 mL). The combined organic extracts were dried with magnesium sulphate, filtered, and the solvent was evaporated in vacuo. Due to the high process yield and purity of the crude products, compound **11a–f** were used in subsequent reactions without further purification.

##### 8-(o-tolylmethyl)-8-azabicyclo[3.2.1]oct-3β-ylamine (**11a**)

Yield: 3.34 g (92.92%) synthesised from 0.02 mol of **10a**.

##### 8-(m-tolylmethyl)-8-azabicyclo[3.2.1]oct-3β-ylamine (**11b**)

Yield: 2.2 g (94.60%) synthesised from 0.012 mol of **10b**.

##### 8-(p-tolylmethyl)-8-azabicyclo[3.2.1]oct-3β-ylamine (**11c**)

Yield: 0.63 g (93.0%) synthesised from 0.003 mol of **10c**.

Compound **11c** was synthesised with minor modification of general procedure described above.

A solution of 0.003 mol of **10c**, 1.1 mL of 10% H_2_SO_4_ and 17.2 mL H_2_O was refluxed with stirring for 4 h. The reaction mixture was cooled to room temperature, washed with 20 mL CH_2_Cl_2_ to remove dark impurities, then the aqueous phase was alkalised with a saturated NaOH (to a pH of 10–12) and extracted with dichloromethane (3 × 20 mL). The combined organic extracts were dried with magnesium sulphate, filtered, and the solvent was evaporated in vacuo. The crude compound **11c** was used in subsequent reactions without further purification.

##### 8-(2-pyridylmethyl)-8-azabicyclo[3.2.1]oct-3β-ylamine **(11d)**

Yield: 2.28 g (91.02%) synthesised from 0.011 mol of **10d**.

##### 8-(3-pyridylmethyl)-8-azabicyclo[3.2.1]oct-3β-ylamine **(11e)**

Synthesised from crude precipitate of **10e**.

##### 8-(4-pyridylmethyl)-8-azabicyclo[3.2.1]oct-3β-ylamine **(11f)**

Yield: 1.86 g (86.51%) synthesised from 0.012 mol of **10f**.

#### General procedure for synthesis of quinoline amides (**12a**–**w**)

A solution of suitable quinolinecarboxylic acid (5 mmol), ethyl chloroformate (0.5 mL, 5 mmol) and triethylamine (0.75 mL, 5 mmol) in anhydrous DMF (25 mL) was stirred for 30 min at 0 °C. A solution of appropriate amine **10a–f** (5 mmol) in anhydrous DMF (15 mL) was added dropwise. The cooling bath was removed and stirring was continued for 24 h. The solvent was evaporated in vacuo and to the residue 10 mL 5% aqueous solution of sodium bicarbonate was added. Next, the aqueous phase was extracted with dichloromethane (3 × 20 mL). The combined organic extracts were dried with magnesium sulphate, filtered, and the solvent was evaporated in vacuo. Final compounds **12a-c**, **12e-s**, and **12v** were purified by crystallisation. Compounds **12d, 12t, 12u**, and **12w** were purified by column chromatography.

##### N-[8-(2-methylbenzyl)-8-azabicyclo[3.2.1]oct-3β-yl)]-quinoline-2-carboxamide (**12a**)

Crystallisation from ethanol. Yield: 0.60 g (77.92%); m.p. 120.8–121.4 °C; ^1^H NMR (500 MHz, CDCl_3_): *δ* s 8.29 (C3”H, C4”H); d 8.11 (C8”H), ^3^*J* = 8.5; d 8.07 (NH), ^3^*J* = 8.5; dd 7.86 (C5”H), ^3^*J* = 8.5, ^4^*J* = 1.0; m 7.76 (C7”H), ^3^*J*_1_ = 8.5, ^3^*J*_2_ = 7.0, ^4^*J* = 1.0; m 7.60 (C6”H), ^3^*J*_1_ = 8.0, ^3^*J*_2_ = 7.0, ^4^*J* = 1.0; m 7.39 (C3’H); m 7.16 (C4’H, C5’H, C6’H); m 4.40 (C3H); s 3.53 (C9H_2_); pt 3.29 (C1H, C5H); s 2.41 (C10H_3_); m 2.11 (C2H(E), C4H(E)); m 1.96 (C6H(E), C7H(E)); pq 1.83 (C6H(A), C7H(A)); td 1.76 (C2H(A), C4H(A)), ^3^*J*_A–A_ = 12.0, ^3^*J*_A–E_ = 2.0; ^13^C NMR (125 MHz, CDCl_3_): *δ* 163.6 (C12); 149.9 (C2”); 146.5 (C8”a); 138.1 (C2’); 137.4 (C4”); 137.1 (C1’); 130.2 (C8”); 130.0 (C7”); 129.7 (C3’); 129.3 (C4”a); 128.9 (C6’); 127.8 (C5”); 127.7 (C6”); 126.7 (C4’); 125.5 (C5’); 118.8 (C3”); 59.2 (C1, C5); 54.5 (C9); 41.8 (C3); 38.9 (C2, C4); 26.6 (C6, C7); 19.3 (C10); IR (KBr) cm^−1^: ν3304 (NH), 1639 (CO); ESI-HRMS *m*/*z* calcd for C_25_H_27_N_3_OH (M + H)^+^ 386.2232, found: 386.2224.

##### N-[8-(2-methylbenzyl)-8-azabicyclo[3.2.1]oct-3β-yl)]-quinoline-3-carboxamide (**12b**)

Crystallisation from ethanol. Yield: 0.50 g (64.93%); m.p. 179.2–180.2 °C; ^1^H NMR (500 MHz, CDCl_3_): *δ* d 9.23 (C2”H), ^4^*J* = 2.0; d 8.52 (C4”H), ^4^*J* = 2.0; d 8.12 (C8”H), ^3^*J* = 8.5; m 7.78 (C7”H), ^3^*J*_1_ = 8.5, ^3^*J*_2_ = 7.0, ^4^*J* = 1.5; m 7.58 (C6”H); m 7.34 (C3’H); m 7.16 (C4’H, C5’H, C6’H); d 6.23 (NH), ^3^*J* = 8.0; m 4.43 (C3H); s 3.50 (C9H_2_); pt 3.27 (C1H, C5H); s 2.38 (C10H_3_); m 2.10 (C2H(E), C4H(E)); m 1.97 (C6H(E), C7H(E)); pq 1.79 (C6H(A), C7H(A)); td 1.67 (C2H(A), C4H(A)), ^3^*J*_A–A_ = 12.0, ^3^*J*_A–E_ = 2.0; ^13^C NMR (125 MHz, CDCl_3_): *δ* 164.9 (C12); 149.2 (C8”a); 148.2 (C2”); 137.9 (C2’); 137.1 (C1’); 135.3 (C4”); 131.1 (C5”); 130.2 (C8”); 129.4 (C3’); 128.8 (C7”); 128.6 (C6’); 127.4 (C4’); 127.3 (C4”a); 126.9 (C3”); 126.7 (C5’); 125.5 (C6”); 59.1 (C1, C5); 54.5 (C9); 42.5 (C3); 39.0 (C2, C4); 26.6 (C6, C7); 19.2 (C10); IR (KBr) cm^−1^: ν3308 (NH), 1627 (CO); ESI-HRMS *m*/*z* calcd for C_25_H_27_N_3_ONa (M + Na)^+^ 408.2052, found: 408.2051.

##### N-[8-(2-methylbenzyl)-8-azabicyclo[3.2.1]oct-3β-yl)]-quinoline-6-carboxamide (**12c**)

Crystallisation from ethyl acetate:ethanol 1:1. Yield: 0.51 g (66.23%); m.p. 211.4–212.7 °C; ^1^H NMR (500 MHz, CDCl_3_): *δ* dd 8.96 (C2”H), ^3^*J* = 4.5, ^4^*J* = 2.0; d 8.20 (C8”H), ^3^*J* = 8.5; d 8.12 (C4”H), ^3^*J* = 9.0; dd 8.02 (C7”H), ^3^*J* = 9.0, ^4^*J* = 2.0; dd 7.44 (C3”H), ^3^*J*_1_ = 8.5, ^3^*J*_2_ = 4.0; m 7.35 (C3’H); m 7.16 (C4’H, C5’H, C6’H); d 6.17 (NH), ^3^*J* = 8.0; m 4.42 (C3H); s 3.51 (C9H_2_); pt 3.27 (C1H, C5H); m 2.10 (C2H(E), C4H(E)); s 2.38 (C10H_3_); m 1.97 (C6H(E), C7H(E)); pq 1.80 (C6H(A), C7H(A)); td 1.66 (C2H(A), C4H(A)), ^3^*J*_A–A_ = 11.5, ^3^*J*_A–E_ = 2.0; ^13^C NMR (125 MHz, CDCl_3_): *δ* 166.1 (C12); 151.9 (C2”); 149.3 (C8”a); 137.9 (C2’); 137.1 (C1’); 136.9 (C5”); 132.7 (C6”); 130.2 (C4”); 129.9 (C3’); 128.8 (C6’); 127.5 (C4”a); 127.1 (C4’); 127.4 (C7”); 126.8 (C5’); 125.5 (C8”); 121.9 (C3”); 59.1 (C1, C5); 54.5 (C9); 42.4 (C3); 39.0 (C2, C4); 26.6 (C6, C7); 19.2 (C10); IR (KBr) cm^−1^: ν3277 (NH), 1620 (CO); ESI-HRMS *m*/*z* calcd for C_25_H_27_N_3_ONa (M + Na)^+^ 408.2052, found: 408.2052.

##### N-[8-(2-methylbenzyl)-8-azabicyclo[3.2.1]oct-3β-yl)]-4-methoxyquinoline-2-carboxamide (**12d**)

Column chromatography chloroform: methanol (9: 1 v/v).Yield: 0.68 g (81.91%); m.p. 54.5–55.9 °C; ^1^H NMR (500 MHz, CDCl_3_): *δ* dd 8.21 (C8”H), ^3^*J* = 8.5, ^4^*J* = 1.0; d 8.02 (C5”H), ^3^*J* = 8.5; m 7.72 (C6”H), ^3^*J*_1_ = 8.0, ^3^*J*_2_ = 6.5, ^4^*J* = 1.5; s 7.69 (C3”H); m 7.54 (C7”H), ^3^*J* = 8.5, ^3^*J*_2_ = 7.0, ^4^*J* = 1.5; m 7.39 (C3’H); m 7.16 (C4’H, C5’H, C6’H); d 6.11 (NH), ^3^*J* = 8.5; m 4.38 (C3H); s 4.11 (OC13H_3_); s 3.53 (C9H_2_); pt 3.29 (C1H, C5H); s 2.41 (C10H_3_); m 2.12 (C2H(E), C4H(E)); m 1.95 (C6H(E), C7H(E)); pq 1.83 (C6H(A), C7H(A)); pt 1.76 (C2H(A), C4H(A)); ^13^C NMR (125 MHz, CDCl_3_): *δ* 163.9 (C12); 163.6 (C4”); 151.4 (C2”); 147.5 (C8”a); 138.1 (C2’); 137.1 (C1’); 130.2 (C7”); 130.2 (C3’); 129.1 (C8”); 128.9 (C6’); 126.7 (C6”); 126.7 (C4’); 125.5 (C5’); 122.0 (C4”a); 121.9 (C5”); 97.8 (C3”); 59.3 (C1, C5); 56.1 (C13); 54.4 (C9); 41.8 (C3); 38.7 (C2, C4); 26.6 (C6, C7); 19.3 (C10); IR (KBr) cm^−1^: ν 3380 (NH), 1674 (CO); ESI-HRMS *m*/*z* calcd for C_26_H_29_N_3_O_2_H (M + H)^+^ 416.2338, found: 416.2325.

##### N-[8-(3-methylbenzyl)-8-azabicyclo[3.2.1]oct-3β-yl)]-quinoline-2-carboxamide (**12e**)

Crystallisation from 2-propanol. Yield: 0.41 g (53.24%); m.p. 113.8–114.7 °C; ^1^H NMR (500 MHz, CDCl_3_): *δ* d 8.29 (C4”H), ^3^*J* = 8.5; d 8.27 (C3”H), ^3^*J* = 8.5; d 8.12 (NH), ^3^*J* = 8.5; d 8.09 (C8”H), ^3^*J* = 8.5; m 7.75 (C7”H), ^3^*J*_1_ = 9.0, ^3^*J*_2_ = 7.0, ^4^*J* = 1.0; dd 7.85 (C5”H), ^3^*J* = 8.0, ^4^*J* = 0.5; m 7.60 (C6”H), ^3^*J*_1_ = 8.0, ^3^*J*_2_ = 7.0, ^4^*J* = 1.0; m 7.22 (C2’H, C5’H, C6’H); m 7.07 (C4’H); m 4.41 (C3H); s 3.55 (C9H_2_); pt 3.30 (C1H, C5H); s 2.36 (C10H_3_); m 2.10 (C2H(E), C4H(E)); m 1.95 (C6H(E), C7H(E)); pq 1.82 (C6H(A), C7H(A)); td 1.78 (C2H(A), C4H(A)), ^3^*J*_A–A_ = 12.5, ^3^*J*_A–E_ = 2.0; ^13^C NMR (125 MHz, CDCl_3_): *δ* 163.6 (C12); 149.9 (C2”); 146.4 (C8”a); 139.9 (C1’); 137.8 (C3’); 137.4 (C4”); 130.0 (C8”); 129.7 (C7”); 129.4 (C6’); 129.2 (C4”a); 128.1 (C5’); 127.8 (C5”); 127.7 (C6”); 127.5 (C2’); 125.7 (C4’); 118.8 (C3”); 58.8 (C1, C5); 56.4 (C9); 41.6 (C3); 38.5 (C2, C4); 26.5 (C6, C7); 21.5 (C10); IR (KBr) cm^−1^: ν3285 (NH), 1647 (CO); ESI-HRMS *m*/*z* calcd for C_25_H_27_N_3_OH (M + H)^+^ 386.2232, found: 386.2241.

##### N-[8-(3-methylbenzyl)-8-azabicyclo[3.2.1]oct-3β-yl)]-quinoline-3-carboxamide (**12f**)

Crystallisation from ethanol. Yield: 0.40 g (51.94%); m.p. 194.0–194.7 °C; ^1^H NMR (500 MHz, CDCl_3_): *δ* d 9.23 (C2”H), ^4^*J* = 2.5; d 8.52 (C4”H), ^4^*J* = 2.0; d 8.13 (C8”H), ^3^*J* = 8.5; dd 7.86 (C5”H), ^3^*J* = 8.0, ^4^*J* = 1.0; m 7.78 (C7”H), ^3^*J*_1_ = 8.0, ^3^*J*_2_ = 6.5, ^4^*J* = 1.5; m 7.59 (C6”H), ^3^*J*_1_ = 8.0, ^3^*J*_2_ = 7.0, ^4^*J* = 1.0; m 7.20 (C5’H), ^3^*J* = 7.5; m 7.19 (C2’H, C6’H); m 7.06 (C4’H), ^3^*J* = 7.5; d 6.24 (NH), ^3^*J* = 8.0; m 4.44 (C3H); s 3.52 (C9H_2_); pt 3.28 (C1H, C5H); s 2.34 (C10H_3_); m 2.09 (C2H(E), C4H(E)); m 1.97 (C6H(E), C7H(E)); pq 1.78 (C6H(A), C7H(A)); td 1.70 (C2H(A), C4H(A)), ^3^*J*_A–A_ = 12.5, ^3^*J*_A–E_ = 2.5; ^13^C NMR (125 MHz, CDCl_3_): *δ* 164.9 (C12); 149.2 (C8”a); 148.2 (C2”); 139.8 (C1’); 137.8 (C3’); 135.3 (C4”); 131.1 (C5”); 129.4 (C8”); 129.3 (C6’); 128.7 (C7”); 128.1 (C5’); 127.6 (C2’); 127.5 (C4’); 127.3 (C3”); 126.9 (C4”a); 125.6 (C6”); 58.8 (C1, C5); 56.3 (C9); 42.4 (C3); 38.6 (C2, C4); 26.5 (C6, C7); 21.4 (C10); IR (KBr) cm^−1^: ν3387 (NH), 1632 (CO); ESI-HRMS *m*/*z* calcd for C_25_H_27_N_3_OH (M + H)^+^ 386.2232, found: 386.2238.

##### N-[8-(3-methylbenzyl)-8-azabicyclo[3.2.1]oct-3β-yl)]-quinoline-6-carboxamide (**12g**)

Crystallisation from 2-propanol. Yield: 0.47 g (61.03 %); m.p. 174.4–175.5 °C; ^1^H NMR (500 MHz, CDCl_3_): *δ* dd 8.97 (C2”H), ^3^*J* = 4.0, ^4^*J* = 2.0; d 8.25 (C5”H), ^4^*J* = 2.0; dd 8.20 (C8”H), ^3^*J* = 8.0, ^5^*J* = 1.0; d 8.13 (C4”H), ^3^*J* = 8.5; dd 8.01 (C7”H), ^3^*J* = 8.5, ^4^*J* = 2.0; dd 7.45 (C3”H), ^3^*J*_1_ = 8.0, ^3^*J*_2_ = 4.0; t 7.21 (C5’H), ^3^*J* = 7.5; m 7.18 (C2’H, C6’H); d 7.07 (C4’H), ^3^*J* = 7.0; d 6.19 (NH), ^3^J = 8.0; m 4.43 (C3H); s 3.53 (C9H_2_); pt 3.29 (C1H, C5H); s 2.35 (C10H_3_); m 2.09 (C2H(E), C4H(E)); m 1.97 (C6H(E), C7H(E)); pq 1.80 (C6H(A), C7H(A)); td 1.70 (C2H(A), C4H(A)), ^3^*J*_A–A_ = 12.0, ^3^*J*_A–E_ = 2.0; ^13^C NMR (125 MHz, CDCl_3_): *δ* 166.0 (C12); 151.9 (C2”); 149.3 (C8”a); 139.8 (C1’); 137.8 (C3’); 136.9 (C5”); 132.7 (C6”); 130.0 (C4”); 129.3 (C6’); 128.1 (C5’); 127.6 (C2’); 127.6 (C4”a); 127.4 (C4’); 127.0 (C7”); 125.6 (C8”); 121.9 (C3”); 58.8 (C1, C5); 56.3 (C9); 42.3 (C3); 38.6 (C2, C4); 26.5 (C6, C7); 21.4 (C10); IR (KBr) cm^−1^: ν3362 (NH), 1632 (CO); ESI-HRMS *m*/*z* calcd for C_25_H_27_N_3_ONa (M + Na)^+^ 408.2052, found: 408.2054.

##### N-[8-(3-methylbenzyl)-8-azabicyclo[3.2.1]oct-3β-yl)]-4-methoxyquinoline-2-carboxamide (**12h**)

Crystallisation from ethyl acetate. Yield: 0.35 g (42.16%); m.p. 116.1–118.3 °C; ^1^H NMR (500 MHz, CDCl_3_): *δ* dd 8.21 (C8”H), ^3^*J* = 8.5, ^4^*J* = 1.0; d 8.16 (NH), ^3^*J* = 8.5; d 8.01 (C5”H), ^3^*J* = 8.5; m 7.72 (C6”H), ^3^*J*_1_ = 8.5, ^3^*J*_2_ = 7.0, ^4^*J* = 1.5; s 7.69 (C3”H); m 7.54 (C7”H), ^3^*J*_1_ = 8.0, ^3^*J*_2_ = 6.5, ^4^*J* = 1.0; m 7.21 (C2’H, C5’H, C6’H); d 7.07 (C4’H), ^3^*J* = 6.5; m 4.39 (C3H); s 4.11 (OC13H_3_); s 3.55 (C9H_2_); pt 3.30 (C1H, C5H); s 2.36 (C10H_3_); m 2.10 (C2H(E), C4H(E)); m 1.94 (C6H(E), C7H(E)); pq 1.82 (C6H(A), C7H(A)); pt 1.79 (C2H(A), C4H(A)); ^13^C NMR (125 MHz, CDCl_3_): *δ* 163.8 (C12); 163.6 (C4”); 151.4 (C2”); 147.5 (C8”a); 139.9 (C1’); 137.8 (C3’); 130.2 (C7”); 129.4 (C6’); 129.1 (C8”); 128.1 (C5’); 127.6 (C2’); 126.7 (C6”); 125.7 (C4’); 122.0 (C4”a); 121.9 (C5”); 97.8 (C3”); 58.8 (C1, C5); 56.3 (C13); 56.1 (C9); 41.7 (C3); 38.4 (C2, C4); 26.6 (C6, C7); 21.4 (C10); IR (KBr) cm^−1^: ν3285 (NH), 1651 (CO); ESI-HRMS *m*/*z* calcd for C_26_H_29_N_3_O_2_H (M + H)^+^ 416.2338, found: 416.2329.

##### N-[8-(4-methylbenzyl)-8-azabicyclo[3.2.1]oct-3β-yl)]-quinoline-2-carboxamide oxalate (**12i**)

Crystallisation from ethanol/diethyl ether 2:1. Yield: 0.53 g (57.6%); m.p. 215.0–215.4 °C; ^1^H NMR (500 MHz, (CD_3_)_2_SO): *δ* d 8.78 (NH); d 8.56 (C4’H), ^3^*J* = 8.5; m 8.15 (C3”H, C8”H); d 8.08 (C5”H), ^3^*J* = 8.0; t 7.87 (C7”H), ^3^*J* = 8.0; t 7.72 (C6”H), ^3^*J* = 8.0; d 7.51 (C3’H, C5’H), ^3^*J* = 7.5; d7.25 (C2’H, C6’H), ^3^*J* = 7.5; m 4.42 (C3H(axial)); s 4.26 (C9H_2_; bs 3.76 (C1H, C5H); s 2.32 (C10H_3_); m 2.20–2.34 C2H(E), C4H(E), C6H(E), C7H(E); m 1.90–2.03 (C2H(A), C4H(A), C6H(A), C7H(A); ^13^C NMR (125 MHz, (CD_3_)_2_SO): *δ* 164.6 (C11); 163.6 (C8”a); 149.9 (C2”); 145.9 (C4”); 138.5 (C1’); 137.5 (C4’); 130.5 (C4”a); 130.4 (C2’, C6’); 129.4 (C3’, C5’); 129.2 (C8’); 128.8 (C7”); 128.2 (C5”); 128.1 (C6”); 59.4 (C1, C5); 51.7 (C9); 39.5 (C3); 33.3 (C2, C4); 24.7 (C6, C7); 20.8 (C10); IR (KBr) cm^−1^: ν3385 (NH), 1688 (CO); ESI-HRMS *m*/*z* calcd for C_25_H_27_N_3_OH (M + H)^+^ 386.2232, found: 386.2216. Based on the analysis of ^1^H and ^13^C NMR spectra, it was found that the resulting compound was monooxolate salt protonated at the 8-position of the tropane.

##### N-[8-(4-methylbenzyl)-8-azabicyclo[3.2.1]oct-3β-yl)]-quinoline-3-carboxamide (**12j**)

Crystallisation from ethyl acetate:ethanol 7:1. Yield: 0.50 g (68.0%); m.p. 219.5–220.8 °C; ^1^H NMR (500 MHz, CDCl_3_): *δ* d 9.23 (C2”H), ^4^*J* = 2.0; d 8.52 (C4”H), ^4^*J* = 2.0; d 8.13 (C8”H), ^3^*J* = 8.5; d 7.86 (C5”H), ^3^*J* = 8.0; m 7.78 (C7”H), ^3^*J*_1_ = 8.5, ^3^*J*_2_ = 6.5, ^4^*J* = 1.0; m 7.59 (C6”H); d 7.26 (C3’H, C5’H), ^3^*J* = 8.0;d 7.12 (C2’H, C6’H), ^3^*J* = 8.0; d 6.21 (NH), ^3^*J* = 8.0; m 4.44 (C3H(axial)), s 3.52 (C9H_2_), bs 3.27 (C1H, C5H); s 2.34 (C10H_3_); m 2.08 (C6H(E), C7H(E); m 1.96 (C2H(E), C4H(E); pq 1.78 (C6H(A), C7H(A); td 1.69 C2H(A), C4H(A), ^3^*J*_A–A_ = 12.0, ^3^*J*_A–E_ = 2.0; ^13^C NMR (125 MHz, CDCl_3_): *δ* 164.9 (C11); 149.2 (C8”a); 148.2 (C2’); 136.8 (C1’); 136.4 (C4’); 135.3 (C4”); 131.1 (C5”); 129.4 (C8”); 128.9 (C2’, C6’); 128.7 (C7’); 128.5 (C3’, C5’); 127.5 (C6”); 127.3 (C3”); 126.9 (C4”a); 58.7 (C1, C5); 56.1 (C9); 42.4 (C3); 38.6 (C2, C4); 26.5 (C6, C7); 21.1 (C10); IR (KBr) cm^−1^: ν3315 (NH), 1632 (CO); ESI-HRMS *m*/*z* calcd for C_25_H_27_N_3_OH (M + H)^+^ 386.2232, found: 386.2241.

##### N-[8-(4-methylbenzyl)-8-azabicyclo[3.2.1]oct-3β-yl)]-quinoline-6-carboxamide (**12k**)

Crystallisation from ethyl acetate:ethanol 1:1. Yield: 0.46 g (59.74%); m.p. 211.9–213.0 °C; ^1^H NMR (500 MHz, CDCl_3_): *δ* dd 8.97 (C2:H), ^3^*J* = 4.0, ^4^*J* = 2.0; d 8.25 (C5”H), ^4^*J* = 2.0; dd 8.20 (C8”H), ^3^*J* = 8.0, ^5^*J* = 1.0; d 8.13 (C4”), ^3^*J* = 8.5; dd 8.01 (C7”H),^3^*J* = 9.0, ^4^*J* = 2.0; dd 7.44 (C3”H) ^3^*J*_1_ = 8.5, ^3^*J*_2_ = 4.0; d 7.27 (C3’H, C5’H), ^3^*J* = 7.5; d 7.12 (C2’H, C6’H), ^4^*J* = 8.0; d 6.18 (NH), ^3^*J* = 8.0; m 4.43 (C3H(axial)), s 3.52 (C9H_2_); bs 3.27 (C1H, C5H); s 2.34 (C10H_3_); m 2.08 (C6H(E)), C7H(E)); m 1.96 (C2H(E), C4H(E)); pq 1.78 (C6H(A), C7H(A)); td 1.67 (C2H(A), C4H(A), ^3^*J*_A-A_ = 12.0, ^3^*J*_A-E_ = 2.5; ^13^C NMR (125 MHz, CDCl_3_): *δ* 166.0 (C11); 151.9 (C2”); 149.3 (C8”a); 136.9 (C5”); 136.9 (C1’); 136.4 (C4’); 132.7 (C6”); 130.0 (C4”); 128.9 (C2’, C6’); 128.5 (C3’, C5’); 127.6 C4”a); 127.4 (C8”); 127.1 (C7”); 121.9 (C3”); 58.7 (C1, C5); 56.1 (C9); 42.4 (C3); 38.7 (C2, C4); 26.5 (C6, C7); 21,1 (C10); IR (KBr) cm^−1^: ν3345 (NH), 1635 (CO); ESI-HRMS *m*/*z* calcd for C_25_H_28_N_3_O (M + H)^+^ 386.2232, found: 386.2240.

##### N-[8-(2-pyridylmethyl)-8-azabicyclo[3.2.1]oct-3β-yl)]-quinoline-2-carboxamide (**12l**)

Crystallisation from ethyl acetate. Yield: 0.26 g (43.34%); m.p. 150.2–151.0 °C; ^1^H NMR (500 MHz, CDCl_3_): *δ* 4d 8.55 (C3’H), ^3^*J* = 5.0, ^4^*J* = 2.0, ^5^*J* = 1.0,; s 8.29 (C4”H, C3”H); d 8.14 (NH), ^3^*J* = 9.0; d 8.10 (C8”H), ^3^*J* = 8.5; 4d 7.86 (C5”H), ^3^*J* = 8.0; m 7.77 (C7”H), ^3^*J*_1_ = 8.5, ^3^*J*_2_ = 7.0, ^4^*J* = 1.5; td 7.68 (C5’H), ^3^*J* = 7.5, ^4^*J* = 2.0; m 7.61 (C6”H, C6’H); 4d 7.16 (C4’H) ^3^*J*_1_ = 7.5, ^3^*J*_2_ = 4.5, ^4^*J* = 1.0; m 4.44 (C3H(axial)); s 3.79 (C9H_2_); pt 3.34 (C1H, C5H); m 2.14 (C2H(E), C4H(E)); m 1.96 (C6H(E), C7H(E)); m 1.86 (C6H(A), C7H(A), C2H(A), C4H(A)); ^13^C NMR (125 MHz, CDCl_3_): *δ* 163.6 (C11); 160.3 (C1’); 149.9 (C2”); 149.0 (C3’); 143.4 (C8”a); 137.4 (C4”); 136.4 (C5’); 130.0 (C8”); 129.7 (C7”); 129.2 (C4”a); 127.8 (C5”); 127.7 (C6”); 122.6 (C6’); 121.8 (C4’); 118.8 (C3”); 59.2 (C1, C5); 58.1 (C9); 41.4 (C3); 37.9 (C2, C4); 26.7 (C6, C7); IR (KBr) cm^−1^: ν3394 (NH), 1679 (CO); ESI-HRMS *m*/*z* calcd for C_23_H_24_N_4_ONa (M + Na)^+^ 395.1848, found: 395.1858.

##### N-[8-(2-pyridylmethyl)-8-azabicyclo[3.2.1]oct-3β-yl)]-quinoline-3-carboxamide (**12m**)

Crystallisation from acetone. Yield: 0.27 g (43.04%); m.p. 169.4–170.5 °C; ^1^H NMR (500 MHz, CDCl_3_): *δ* d 9.24 (C2”H), ^4^*J* = 2.0;m 8.53 (C3’H, C4”H); 4d 8.13 (C8”H), ^3^*J* = 8.5; 4d 7.87 (C5”H), ^3^*J* = 8.0, ^4^*J* = 1.0, ^5^*J* = 0.5; m 7.78 (C7”H), ^3^*J*_1_ = 8.5, ^3^*J*_2_ = 6.5, ^4^*J* = 1.5; td 7.66 (C5’H), ^3^*J* = 8.0, ^4^*J* = 2.0; m 7.60 (C6”H), ^3^*J*_1_ = 8.0, ^3^*J*_2_ = 7.0, ^4^*J* = 1.0; dt 7.55 (C6’H), ^3^*J* = 6.5, ^4^*J* = ^5^*J* = 1.0; 4d 7.16 (C4’H), ^3^*J*_1_ = 7.0, ^3^*J*_2_ = 5.0, ^4^*J* = 1.5; d .31 (NH), ^3^*J* = 8.0; m 4.46 (C3H(axial)); s 3.74 (C9H_2_); pt 3.31 (C1H, C5H); m 2.13 (C2H(E), C4H(E)); m 1.98 (C6H(E), C7H(E)); pk 1.84 (C6H(A), C7H(A)); td 1.77 (C2H(A), C4H(A)), ^2,3^*J*_A–A_ = 11.5, ^3^*J*_A–E_ = 2.0; ^13^C NMR (125 MHz, CDCl_3_): *δ* 164.9 (C11); 160.1 (C1’); 149.2 (C8”a); 149.0 (C3’); 148.2 (C2”); 136.4 (C5’); 135.3 (C4”); 131.1 (C5”); 129.3 (C8”); 128.7 (C7”); 127.4 (C6”); 127.2 (C3”); 126.9 (C4”a); 122.6 (C6’); 121.9 (C4’); 59.1 (C1, C5); 58.1 (C9); 42.2 (C3); 38.0 (C2, C4); 26.7 (C6, C7); IR (KBr) cm^−1^: ν3283 (NH), 1631 (CO); ESI-HRMS *m*/*z* calcd for C_23_H_24_N_4_ONa (M + Na)^+^ 395.1848, found: 395.1857.

##### N-[8-(2-pyridylmethyl)-8-azabicyclo[3.2.1]oct-3β-yl)]-quinoline-6-carboxamide (**12n**)

Crystallisation from acetone. Yield: 0.50 g (83.35%); m.p. 193.7–194.5 °C; ^1^H NMR (500 MHz, CDCl_3_): *δ* dd 8.97 (C2”H), ^3^*J* = 4.0, ^4^*J* = 1.5; dd 8.54 (C3’H), ^3^*J* = 4.5, ^4^*J* = 1.0; d 8.25 (C5”H), ^4^*J* = 1.5; dd 8.20 (C8”H), ^3^*J* = 8.5, ^5^*J* = 0.5; d 8.13 (C4”H), ^3^*J* = 9.0; dd 8.02 (C7”H), ^3^*J* = 9.0, ^4^*J* = 2.0; dd 7.45 (C3”H), ^3^*J*_1_ = 8.5, ^3^*J*_2_ = 4.0; td 7.66 (C5’H), ^3^*J* = 7.5, ^4^*J* = 2.0; d 7.55 (C6’H), ^3^*J* = 7.5; td 7.16 (C4’H), ^3^*J* = 6.5; d 6.24 (NH), ^3^*J* = 8.5; m 4.45 (C3H(axial)); s 3.75 (C9H_2_); pt 3.31 (C1H, C5H); m 2.13 (C2H(E), C4H(E)); m 1.98 (C6H(E), C7H(E)); pk 1.82 (C6H(A), C7H(A)); td 1.76 (C2H(A), C4H(A)), ^2,3^*J*_A–A_ = 12.0, ^3^*J*_A–E_ = 2.0; ^13^C NMR (125 MHz, CDCl_3_): *δ* 166.0 (C11); 160.1 (C1’); 151.9 (C2”); 149.3 (C8”a); 149.0 (C3’); 136.9 (C5”); 136.7 (C6”); 136.4 (C5’); 129.9 (C4”); 127.5 (C4”a); 127.4 (C7”); 127.0 (C8”); 122.5 (C6’); 121.9 (C3”); 121.9 (C4’); 59.1 (C1, C5); 58.1 (C9); 42.1 (C3); 38.0 (C2, C4); 26.7 (C6, C7); IR (KBr) cm^−1^: ν3268 (NH), 1635 (CO); ESI-HRMS *m*/*z* calcd for C_23_H_24_N_4_ONa (M + Na)^+^ 395.1848, found 395.1848.

##### N-[8-(2-pyridylmethyl)-8-azabicyclo[3.2.1]oct-3β-yl)]-4-methoxyquinoline-2-carboxamide (**12o**)

Crystallisation from diisopropyl ether. Yield: 0.57 g (75.06%); m.p. 132.5–133.0 °C; ^1^H NMR (500 MHz, CDCl_3_): *δ* 4d 8.54 (C3’H), ^3^*J* = 4.5, ^4^*J* = 1.5, ^5^*J* = 0.5; 4d 8.21 (C8”H), ^3^*J* = 8.5, ^4^*J* = 1.5, ^5^*J* = 1.0; d 8.18 (NH), ^3^*J* = 9.0; 4d 8.02 (C5”H), ^3^*J* = 8.5, ^4^*J* = 1.5, ^5^*J* = 1.0; m 7.73 (C6”H), ^3^*J*_1_ = 8.5, ^3^*J*_2_ = 7.0, ^4^*J* = 1.5; s 7.69 (C3”H); m 7.55 (C7”H), ^3^*J*_1_ = 8.5, ^3^*J*_2_ = 7.0, ^4^*J* = 1.5; td 7.68 (C5’H), ^3^*J* = 7.5, ^4^*J* = 1.5; d 7.60 (C6’H), ^3^*J* = 7.5; 4d 7.16 (C4’H), ^3^*J*_1_ = 7.5, ^3^*J*_2_ = 5.0, ^4^*J* = 1.5; m 4.41 (C3H(axial)); s 4.12 (OCH_3_); s 3.78 (C9H_2_); pt 3.33 (C1H, C5H); m 2.14 (C2H(E), C4H(E)); pk 1.82 (C6H(A), C7H(A), C2H(A), C4H(A)); m 1.46 (C6H(E), C7H(E)); ^13^C NMR (125 MHz, CDCl_3_): *δ* 163.8 (C11); 163.5 (C4”); 160.3 (C1’); 151.4 (C2”); 149.0 (C3’); 147.5 (C8”a); 136.4 (C5’); 130.2 (C7”); 129.1 (C8”); 126.7 (C6”); 122.6 (C6’); 122.0 (C4”a); 121.9 (C5”); 121.8 (C4’); 97.8 (C3”); 59.2 (C1, C5); 58.2 (C9); 56.1 (OCH_3_); 41.5 (C3); 37.9 (C2, C4); 26.8 (C6, C7); IR (KBr) cm^−1^: ν3372 (NH), 1671 (CO); ESI-HRMS *m*/*z* calcd for C_24_H_26_N_4_O_2_Na (M + Na)^+^ 425.1953, found: 425.1948.

##### N-[8-(3-pyridylmethyl)-8-azabicyclo[3.2.1]oct-3β-yl)]-quinoline-2-carboxamide (**12p**)

Crystallisation from ethyl acetate. Yield: 0.52 g (69%); m.p. 143.0–143.4 °C; ^1^H NMR (500 MHz, CDCl_3_): *δ* d 8.61 (C2’H), ^4^*J* = 1.5; dd 8.51 (C4’H), ^3^*J* = 4.5, ^4^*J* = 1.5; s 8.29 (C3”H, C4”H); bs 8.13 (NH); d 8.10 (C8”H), ^3^*J* = 8.5; 4d 7.86 (C5”H), ^3^*J* = 8.5, ^4^*J* = 1.5, ^5^*J* = 0.5; dt 7.78 (C6’H), ^3^*J* = 7.5; m 7.76 (C7”H), ^3^*J*_1_ = 8.5, ^3^*J*_2_ = 7.0, ^4^*J* = 1.5; m 7.61 (C6”H), ^3^*J*_1_ = 8.0, ^3^*J*_2_ = 6.5, ^4^*J* = 1.0; 4d 7.27 (C5’H), ^3^*J*_1_ = 8.0, ^3^*J*_2_ = 4.5, ^5^*J* = 0.5; m 4.41 (C3H(axal)); s 3.59 (C9H_2_); pt 3.28 (C1H, C5H); m 2.10 (C2H(E), C4H(E)); m 1.96 (C6H(E), C7H(E)); pk 1.84 (C6H(A), C7H(A)); td 1.78 (C2H(A), C4H(A)), ^3^*J*_A–A_ = 12.5, ^3^*J*_A–E_ = 2.0; ^13^C NMR (125 MHz, CDCl_3_): *δ* 163.6 (C11); 150.0 (C2’); 149.8 (C2”); 148.4 (C4’); 146.4 (C8”a); 137.4 (C4”); 135.4 (C1’); 135.4 (C6’); 130.0 (C8”); 129.6 (C7”); 129.2 (C4”a); 127.8 (C5”); 127.7 (C6”); 123.3 (C5’); 118.8 (C3”); 58.9 (C1, C5); 53.7 (C9); 41.4 (C3); 38.3 (C2, C4); 26.5 (C6, C7); IR (KBr) cm^−1^: ν3369 (NH), 1678 (CO); ESI-HRMS *m*/*z* calcd for C_23_H_24_N_4_ONa (M + Na)^+^ 395.1848, found: 395.1838.

##### N-[8-(3-pyridylmethyl)-8-azabicyclo[3.2.1]oct-3β-yl)]-quinoline-3-carboxamide (**12q**)

Crystallisation from ethyl acetate. Yield: 0.52 g (69%); m.p. 138.3–146.2 °C; ^1^H NMR (500 MHz, CDCl_3_): *δ* d 9.25 (C2”H), ^4^*J* = 2.0; d 8.59 (C2’H), ^4^*J* = 1.5; d 8.56 (C4”H), ^4^*J* = 2.0; dd 8.50 (C4’H), ^3^*J* = 5.0, ^4^*J* = 2.0; dd 8.13 (C8”H), ^3^*J* = 8.5, ^4^*J* = 1.0; dd 7.88 (C5”H), ^3^*J* = 8.0, ^4^*J* = 1.5; m 7.79 (C7”H), ^3^*J*_1_ = 8.5, ^3^*J*_2_ = 7.0, ^4^*J* = 1.5; dt 7.74 (C6’H), ^3^*J* = 8.0, ^4^*J* = 2.0; m 7.60 (C6”H), ^3^*J*_1_ = 8.5, ^3^*J*_2_ = 7.0, ^4^*J* = 1.5; 4d 7.26 (C5’H), ^3^*J*_1_ = 8.0, ^3^*J*_2_ = 5.0, ^5^*J* = 0.5; d 6.45 (NH), ^3^*J* = 8.5; m 4.45 (C3H(axial)); s 3.57 (C9H_2_); pt 3.26 (C1H, C5H); m 2.11 (C2H(E), C4H(E)); m 1.98 (C6H(E), C7H(E)); pk 1.83 (C6H(A), C7H(A)); td 1.71 (C2H(A), C4H(A)), ^3^*J*_A–A_ = 11.0, ^3^*J*_A–E_ = 2.5; ^13^C NMR (125 MHz, CDCl_3_): *δ* 165.0 (C11); 150.0 (C2’); 149.2 (C8”a); 148.4 (C4’); 148.2 (C2”); 136.3 (C6’); 135.4 (C4”); 135.3 (C1’); 131.2 (C5”); 129.3 (C8”); 128.7 (C7”); 127.5 (C6”); 127.2 (C3”); 126.9 (C4”a); 123.4 (C5’); 59.0 (C1, C5); 53.8 (C9); 42.2 (C3); 38.5 (C2, C4); 26.4 (C6, C7); IR (KBr) cm^−1^: ν3250 (NH), 1653 (CO); ESI-HRMS *m*/*z* calcd for C_23_H_24_N_4_ONa (M + Na)^+^ 395.1848, found: 395.1844.

##### N-[8-(3-pyridylmethyl)-8-azabicyclo[3.2.1]oct-3β-yl)]-quinoline-6-carboxamide (**12r**)

Crystallisation from ethyl acetate. Yield: 0.43 g (57%); m.p. 158.3–162.5 °C; ^1^H NMR (500 MHz, CDCl_3_): *δ* dd 8.93 (C2”H), ^3^*J* = 4.0, ^4^*J* = 2.0; dd 8.58 (C2’H), ^4^*J*_1_ = 2.0, ^4^*J*_2_ = 1.0; dd 8.49 (C4’H), ^3^*J* = 4.5, ^4^*J* = 2.0; d 8.29 (C5”H), ^4^*J* = 2.0; 4d 8.18 (C8”H), ^3^*J* = 8.5, ^5^*J* = 1.0, ^p^*J* = 0.5; d 8.11 (C4”H), ^3^*J* = 8.5; dd 8.06 (C7”H), ^3^*J* = 8.5, ^4^*J* = 2.0; dt 7.71 (C6’H), ^3^*J* = 8.0, ^4^*J* = 2.0; dd 7.43 (C3”H), ^3^*J*_1_ = 8.5, ^3^*J*_2_ = 4.0; 4d 7.25 (C5’H), ^3^*J*_1_ = 8.0, ^3^*J*_2_ = 4.5, ^5^*J* = 1.0; d 6.73 (NH), ^3^*J* = 8.5; m 4.43 (C3H(axial)); s 3.55 (C9H_2_); pt 3.24 (C1H, C5H); m 2.08 (C2H(E), C4H(E)); m 1.95 (C6H(E), C7H(E)); pk 1.81 (C6H(A), C7H(A)); td 1.70 (C2H(A), C4H(A)), ^3^*J*_A–A_ = 12.5, ^3^*J*_A–E_ = 2.0; ^13^C NMR (125 MHz, CDCl_3_): *δ* 166.2 (C11); 151.8 (C2”); 149.9 (C2’); 149.1 (C8”a); 148.2 (C4’); 136.9 (C5”); 136.3 (C6’); 135.3 (C1’); 132.7 (C6”); 129.7 (C4”); 127.5 (C7”); 127.5 (C4”a); 127.2 (C8”); 123.3 (C5’); 121.8 (C3”); 58.9 (C1, C5); 53.8 (C9); 42.1 (C3); 38.3 (C2, C4); 26.4 (C6, C7); IR (KBr) cm^−1^: ν3233 (NH), 1628 (CO); ESI-HRMS *m*/*z* calcd for C_23_H_24_N_4_ONa (M + Na)^+^ 395.1848, found 395.1839.

##### N-[8-(3-pyridylmethyl)-8-azabicyclo[3.2.1]oct-3β-yl)]-4-methoxyquinoline-2-carboxamide (**12s**)

Crystallisation from acetone. Yield: 0.55 g (68%); m.p. 162.6–165.5 °C; ^1^H NMR (500 MHz, CDCl_3_): *δ* d 8.61 (C2’H), ^4^*J* = 1.5; dd 8.51 (C4’H), ^3^*J* = 4.5, ^4^*J* = 1.5; dd 8.21 (C8”H), ^3^*J* = 8.5, ^4^*J* = 1.0; d 8.16 (NH), ^3^*J* = 8.5; 4d 8.02 (C5”H), ^3^*J* = 8.0, ^4^*J* = ^5^*J* = 1.0; dt 7.78 (C6’H), ^3^*J* = 7.5, ^4^*J* = 2.0; m 7.73 (C6”H), ^3^*J*_1_ = 8.5, ^3^*J*_2_ = 6.5, ^4^*J* = 1.5; s 7.69 (C3”H); m 7.55 (C7”H), ^3^*J*_1_ = 8.5, ^3^*J*_2_ = 7.0, ^4^*J* = 1.5; 4d 7.27 (C5’H), ^3^*J*_1_ = 7.5, ^3^*J*_2_ = 4.5, ^5^*J* = 0.5; m 4.39 (C3H(axial)); s 4.11 (OCH_3_); s 3.60 (C9H_2_); pt 3.27 (C1H, C5H); m 2.10 (C2H(E), C4H(E)); m 1.95 (C6H(E), C7H(E)); pk 1.84 (C6H(A), C7H(A)); td 1.78 (C2H(A), C4H(A)), ^3^*J*_A–A_ = 12.0, ^3^*J*_A–E_ = 2.0; ^13^C NMR (125 MHz, CDCl_3_): *δ* 163.9 (C11); 163.5 (C4”); 151.3 (C2”); 150.0 (C2’); 148.4 (C4’); 147.5 (C8”a); 136.2 (C6’); 135.4 (C1’); 130.2 (C7”); 129.1 (C8”); 126.7 (C6”); 123.3 (C5’); 122.0 (C4”a); 121.9 (C5”); 97.7 (C3”); 58.9 (C1, C5); 56.1 (OCH_3_), 53.7 (C9); 41.5 (C3); 38.2 (C2, C4); 26.5 (C6, C7); IR (KBr) cm^−1^: ν3326 (NH), 1663 (CO); ESI-HRMS *m*/*z* calcd for C_24_H_26_N_4_O_2_Na (M + Na)^+^ 425.1953, found: 425.1934.

##### N-[8-(4-pyridylmethyl)-8-azabicyclo[3.2.1]oct-3β-yl)]-quinoline-2-carboxamide (**12t**)

Column chromatography chloroform: methanol (98: 2 v/v). Yield: 0.46 g (61.74%); m.p. 143.0–145.7 °C; ^1^H NMR (500 MHz, CDCl_3_): *δ* dd 8.56 (C2’H, C6’H), ^3^*J* = 5.5, ^4^*J* = 1.5; s 8.30 (C3”H, C4”H); pd 8.11 (C8”H, NH); dd 7.87 (C5”H), ^3^*J* = 8.0, ^4^*J* = 0.5; m 7.77 (C7”H), ^3^*J*_1_ = 8.5, ^3^*J*_2_ = 7.0, ^4^*J* = 1.5; m 7.61 (C6”H); d 7.37 (C3’H, C5’H), ^3^*J* = 5.5; m 4.42 (C3H(axial)), ^3^*J*_A–A_ = 13.0, ^3^*J*_A–E_ = 9.0; s 3.60 (C9H_2_); pt 3.27 (C1H, C5H); m 2.09 (C2H(E), C4H(E)); m 1.98 (C6H(E), C7H(E)); pq 1.85 (C6H(A), C7H(A)); td 1.81 (C2H(A), C4H(A)), ^3^*J*_A–A_ = 11.5, ^3^*J*_A–E_ = 2.0; ^13^C NMR (125 MHz, CDCl_3_): *δ* 163.7 (C11); 149.8 (C2”); 149.7 (C2’, C6’); 149.4 (C4’); 146.5 (C8”a); 137.4 (C4”); 130.1 (C8”); 129.6 (C7”); 129.3 (C4”a); 127.8 (C5”); 127.7 (C6”); 123.4 (C3’, C5’); 118.8 (C3”); 59.3 (C1, C5); 55.4 (C9); 41.4 (C3); 38.4 (C2, C4); 26.6 (C6, C7); IR (KBr) cm^−1^: ν3277 (NH), 1659 (CO); ESI-HRMS *m*/*z* calcd for C_23_H_24_N_4_ONa (M + Na)^+^ 395.1848, found: 395.1835.

##### N-[8-(4-pyridylmethyl)-8-azabicyclo[3.2.1]oct-3β-yl)]-quinoline-3-carboxamide (**12u**)

Column chromatography chloroform: methanol (95: 5 v/v). Yield: 0.44 g (58.34%); m.p. 69.5–72.0 °C; ^1^H NMR (500 MHz, CDCl_3_): *δ* *C3H axial conformation:* d 9.27 (C2”H), ^4^*J* = 2.5; d 8.58 (C4”H), ^4^*J* = 2.0; m* 8.54 (C2’H, C6’H); m* 8.14 (C8”H); d 7.86 (C5”H), ^3^*J* = 8.0; m* 7.79 (C7”H); m* 7.60 (C6”H); m* 7.34 (C3’H, C5’H); d 6.67 (NH), ^3^*J* = 8.0; m 4.46 (C3H); s* 3.57 (C9H_2_); m* 3.25 (C1H, C5H); m 2.09 (C2H(E), C4H(E)); m 2.00 (C6H(E),C7H(E)); m* 1.84 (C6H(A), C7H(A)); td 1.75 (C2H(A), C4H(A), ^3^*J*_A–A_ = 12.5, ^3^*J*_A–E_ = 2.5, *C3H equatorial conformation:* d 9.21 (C2”H), ^4^*J* = 2.0; m* 8.54 (C4”H); m* 8.54 (C2’H, C6’H); m* 8.14 (C8”H); d 7.90 (C5”H), ^3^*J* = 7.5; m* 7.80 (C7”H); m* 7.60 (C6”H); m* 7.34 (C3’H, C5’H); d 6.75 (NH), ^3^*J* = 6.5; pq 4.40 (C3H); s* 3.57 (C9H_2_); m* 3.25 (C1H, C5H); m 2.35 (C6H(E), C7H(E)); m 2.23 (C2H(E), C4H(E)); pq 1.94 (C6H(A), C7H(A)); m* 1.84 (C2H(A), C4H(A)); ^13^C NMR (125 MHz, CDCl_3_): *δ* *C3H axial conformation:* 165.1 (C11); 149.6 (C2’, C6’); 149.3 (C4’); 149.1 (C8”a); 148.3 (C2”); 135.4 (C4”); 131.1 (C5”); 129.3 (C8”); 128.7 (C7”); 127.5 (C6”); 127.3 (C3”); 126.9 (C4”a); 123.4 (C3’, C5’); 59.4 (C1, C5); 55.5 (C9); 42.1 (C3); 38.5 (C2, C4); 26.5 (C6, C7), *C3H equatorial conformation:* 164.7 (C11); 149.7 (C2’, C6’); 149.2 (C4’); 149.2 (C8”a); 147.7 (C2”); 135.5 (C4”); 131.2 (C5”); 129.3 (C8”); 128.7 (C7”); 127.6 (C6”); 127.4 (C3”); 126.9 (C4”a); 123.3 (C3’, C5’); 58.4 (C1, C5); 55.7 (C9); 42.6 (C3); 36.7 (C2, C4); 26.5 (C6, C7), *-there is a increase in the number of signals in the ^1^H NMR spectra, suggesting the presence of a mixture of compounds. distinct multiplets of the C3H proton (in the proton spectrum) allows the conclusion that there is a mixture of two isomers: one (β) with an axial multiplet of the C3H proton (12 lines) and one (α) with an equatorial multiplet of the C3H proton (4 lines). The molar ratio of the β form to the α form is 2:1; IR (KBr) cm^−1^: ν3292 (NH), 1636 (CO); ESI-HRMS *m*/*z* calcd for C_23_H_24_N_4_ONa (M + Na)^+^ 395.1848, found: 395.1844.

##### N-[8-(4-pyridylmethyl)-8-azabicyclo[3.2.1]oct-3β-yl)]-quinoline-6-carboxamide (**12v**)

Crystallisation from acetone. Yield: 0.31 g (41.61%); m.p. 182.0–183.3 °C; ^1^H NMR (500 MHz, CDCl_3_): *δ* dd 8.97 (C2”H), ^3^*J* = 4.0, ^4^J = 1.5; d 8.54 (C2’H, C6’H), ^3^*J* = 5.5; d 8.29 (C5”H), ^4^J = 2.0; dd 8.20 (C8”H), ^3^*J* = 8.0, ^5^*J* = 1.0; d 8.13 (C4”H), ^3^*J* = 9.0; dd 8.06 (C7”H), ^3^*J* = 8.5, ^4^*J* = 2.0; dd 7.45 (C3”H), ^3^*J*_1_ = 8.5, ^3^*J*_2_ = 4.5; d 7.33 (C3’H, C5’H), ^3^*J* = 5.5; d 6.52 (NH), ^3^*J* = 8.0; m 4.44 (C3H); s 3.56 (C9H_2_); pt 3.24 (C1H, C5H); m 2.08 (C2H(E), C4H(E)); m 1.99 (C6H(E), C7H(E)); pq 1.82 (C6H(A), C7H(A)); td 1.73 (C2H(A), C4H(A)), ^3^*J*_A–A_ = 12.5, ^3^*J*_A–E_ = 2.5; ^13^C NMR (125 MHz, CDCl_3_): *δ* 166.2 (C11); 151.9 (C2”); 149.6 (C2’, C6’); 149.5 (C4’); 149.3 (C8”a); 136.9 (C5”); 132.7 (C6”); 129.9 (C4”); 127.5 (C4”a); 127.5 (C7”); 127.1 (C8”); 123.3 (C3’, C5’); 121.9 (C3”); 59.4 (C1, C5); 55.5 (C9); 42.0 (C3); 38.6 (C2, C4); 26.5 (C6, C7); IR (KBr) cm^−1^: ν3277 (NH), 1647 (CO); ESI-HRMS *m*/*z* calcd for C_23_H_24_N_4_ONa (M + Na)^+^ 395.1848, found 395.1832.

##### N-[8-(4-pyridylmethyl)-8-azabicyclo[3.2.1]oct-3β-yl)]-4-methoxyquinoline-2-carboxamide (**12w**)

Column chromatography chloroform: methanol (96: 4 v/v). Yield: 0.40 g (49.19%); m.p. 52.0–55.0 °C; ^1^H NMR (500 MHz, CDCl_3_): *δ* dd 8.55 (C2’H, C6’H), ^3^*J* = 4.5, ^4^*J* = 1.5; dd 8.22 (C8”H), ^3^*J* = 8.5, ^4^*J* = 1.5; d 8.17 (NH), ^3^*J* = 8.5; d 8.03 (C5”H), ^3^*J* = 8.5; m 7.73 (C6”H), ^3^*J*_1_ = 9.0, ^3^*J*_2_ = 6.5, ^4^*J* = 1.5; s 7.69 (C3”H); m 7.55 (C7”H), ^3^*J*_1_ = 8.5, ^3^*J*_2_ = 7.0, ^4^*J* = 1.0; d 7.36 (C3’H, C5’H), ^3^*J* = 6.0; m 4.39 (C3H(axial)); s 4.12 (OCH_3_); s 3.60 (C9H_2_); pt 3.26 (C1H, C5H); m 2.09 (C2H(E), C4H(E)); m 1.97 (C6H(E), C7H(E)); pq 1.85 (C6H(A), C7H(A)); td 1.81 (C2H(A), C4H(A)), ^3^*J*_A–A_ = 12.5, ^3^*J*_A–E_ = 2.5; ^13^C NMR (125 MHz, CDCl_3_): *δ* 163.9 (C11); 163.6 (C4”); 151.3 (C2”); 149.7 (C2’, C6’); 149.4 (C4’); 147.5 (C8”a); 130.3 (C7”); 129.1 (C8”); 126.8 (C6”); 123.4 (C3’, C5’); 122.0 (C4”a); 122.0 (C5”); 97.8 (C3”); 59.3 (C1, C5); 56.1 (C12); 55.3 (C9); 41.5 (C3); 38.2 (C2, C4); 26.6 (C6, C7); IR (KBr) cm^−1^: ν3323 (NH), 1670 (CO); ESI-HRMS *m*/*z* calcd for C_24_H_26_N_4_O_2_Na (M + Na)^+^ 425.1953, found: 425.1965.

#### General procedure for synthesis of N-[8-(nitrobenzyl)-8-azabicyclo[3.2.1]oct-3β-yl)]-2-naphthamides (**18a**–**c**)

N-(8-azabicyclo[3.2.1]oct-3β-yl)-2-naphthamide hydrochloride (**17**) 1.38 g (4.4 mmol), 0.76 g (4.4 mmol) of an appropriate nitrobenzyl chloride (2-nitrobenzyl chloride for **18a**, 3-nitrobenzyl chloride for **18b**, 4-nitrobenzyl chloride for **18c**), K_2_CO_3_ 1.38 g (10.0 mmol) and 100 mg of KI were suspended in 60 mL of acetone. The reaction mixture was refluxed with stirring. The reaction time was determined using TLC. Solvent was removed and the residue was dissolved in a mixture of 30 mL of CH_3_Cl and 30 mL of water. The aqueous phase was extracted with CH_3_Cl (2 × 20 mL). The combined organic extracts were dried with magnesium sulphate, filtered, and the solvent was evaporated in vacuo. Compounds **18a–c** were purified by crystallisation.

##### N-[8-(2-nitrobenzyl)-8-azabicyclo[3.2.1]oct-3β-yl)]-2-naphthamide (**18a**)

Crystallisation from anhydrous ethanol. Yield: 1.04 g (55.0%); m.p. 177.7-179.6 °C; ^1^H NMR (500 MHz, CDCl_3_): *δ* s 8.24 (C1”H); dd 7.91 (C3’H), ^3^*J* = 7.5; m 7.86 (C3”H, C8”H); m 7.81 (C4”H, C5”H); d 7.65 (C6’H), ^3^*J* = 7.5; m 7.50–7.58 (C5’H, C6”H, C7”H); td 7.38 (C4’H), ^3^*J* = 8.0, ^4^*J* = 1.0; d 6.07 (NH), ^3^*J* = 8.5; m 4.39 (C3H(axial)); s 3.83 (C9H_2_), pt 3.18 (C1H, C5H; m 2.05 (C6H(E), C7H(E)); m 1.94 (C2H(E), C4H(E)); pq 1.80 (C6H(A), C7H(A)); td 1.60 (C2H(A), C4H(A), ^3^*J*_A–A_ = 12.0, ^3^*J*_A–E_ = 2.5; ^13^C NMR (125 MHz, CDCl_3_): *δ* 166.8 (C11); 149.9 (C2’); 135.4 (C4”a); 134.7 (C2”); 132.6 (C1’); 132.3 (C5’); 132.0 (C8”a); 130.5 (C8”); 128.9 (C5”); 128.4 (C1”); 127.7 (C4’, C4”); 127.7 (C6’); 127.6 (C7”); 127.2 (C6”); 126.7 (C3”); 124.3 (C3’); 123.6 (C1’); 59.6 (C1, C5); 53.6 (C9); 41.9 (C3); 38.7 (C2, C4); 26.6 (C6, C7); IR (KBr) cm^-1^: ν3252 (NH), 1631 (CO), 1551 (C-NO_2_ *asym*); 1334 (C-NO_2_ *sym*); ESI-HRMS *m*/*z* calcd for C_25_H_25_N_3_O_3_Na (M + Na)^+^ 438.1794, found 438.1801.

##### N-[8-(3-nitrobenzyl)-8-azabicyclo[3.2.1]oct-3β-yl)]-2-naphthamide (**18b**)

Crystallisation from anhydrous ethanol. Yield: 0.97 g (51.3%); m.p. 184.5–185.8 °C; ^1^H NMR (500 MHz, CDCl_3_): *δ* s 8.34 (C2’H); s 8.26 (C1”H); dd 8.10 (C4’H), ^3^*J* = 8.0, ^4^*J* = 1.0; m 7.94–7.80 (C3”H, C4:H, C”H, C8”H); d 7.69 (C6’H), ^3^*J* = 7.5; m 7.54 (C6”H, C7”H0; t 7.46 (C5’H), ^3^*J* = 7.5; d 6.17 (NH), ^3^*J* = 8.5; m 4.44 (C3H(axial)); s 3.65 (C9H_2_), bs 3.24 (C1H, C5H); m 2.08 (C6H(E), C7H(E)); m 2.00 (C2H(E), C4H(E)); pq 1.83 (C6H(A), C7H(A)); td 1.71 (C2H(A), C4H(A)), ^3^*J*_A–A_ = 12.0, ^3^*J*_A–E_ = 2.0; ^13^C NMR (125 MHz, CDCl_3_): *δ* 166.8 (C11); 148.4 (C3’); 142.6 (C1’); 134.7 (C4”a); 134.4 (C6’); 132.6 (C2”); 131.9 (C8”a); 129.0 (C8”); 128.9 (C5”); 128.4 (C5’); 127.7 (C1”); 127.6 (C4”); 127.2 (C7”); 126.7 (C6”); 123.6 (C3”); 123.2 ()C4’); 121.9 (C2’); 59.2 (C1, C5); 55.8 (C9); 42.0 (C3); 38.7 (C2, C4); 26.5 (C6, C7); IR (KBr) cm^−1^: ν3257 (NH), 1637 (CO), 1554 (C–NO_2_ *asym*), 1346 (C–NO_2_ *sym*); ESI-HRMS *m*/*z* calcd for C_25_H_25_N_3_O_3_Na (M + Na)^+^ 438.1794, found 438.1801.

##### N-[8-(4-nitrobenzyl)-8-azabicyclo[3.2.1]oct-3β-yl)]-2-naphthamide (**18c**)

Crystallisation from acetone. Yield: 1.32 g (69.8%); m.p. 207.0–208.6 °C;^1^H NMR (500 MHz, CDCl_3_): *δ* s 8.26 (C1”H; d 8.16 (C3’H, C5’H), ^3^*J* = 8.5; m 7.91–7.80 (C3”H, C4”H, C5”H, C8”H); m 7.60–7.50 (C2’H, C6’H, C6”H, C7”H); d 6.17 (NH), ^3^*J* = 8.0; m 4.43 (C3H(axial)); s 3.65 (C9H_2_); ps 3.23 (C1H, C5H); m 2.07 (C6H(E), C7H(E)); m 2.00 (C2H(E),C4H(E)); pq 1.83 (C6H(A), C7H(A)); m 1.70 (C2H(A), C4H(A)); ^13^C NMR (125 MHz, CDCl_3_): *δ* 166.8 (C11); 148.1 (C4’); 147.0 (C1’); 134.7 (C4”a); 132.6 (C2”); 131.9 (C8”a); 128.9 C2’, C6’); 128.8 (C8”); 128.4 (C5”); 127.7 (C1”); 127.6 (C4”); 127.2 (C7”); 126.8 (C6”); 123.5 (C3”); 123.5 (C3’, C5’); 59.3 (C1, C5); 55.9 (C9); 42.0 (C3); 38.7 (C2, C4); 26.5 (C6, C7); IR (KBr) cm^−1^: ν3251 (NH), 1634 (CO), 1555 (C–NO_2_ *asym*); 1345 (C–NO_2_ *sym*); ESI-HRMS *m*/*z* calcd for C_25_H_25_N_3_O_3_Na (M + Na)^+^ 438.1794, found 438.1784.

#### General procedure for synthesis of N-[8-(aminobenzyl)-8-azabicyclo[3.2.1]oct-3β-yl)]-2-naphthamides (**19a**–**c**)

The appropriate *N-[8-(nitrobenzyl)-8-azabicyclo[3.2.1]oct-3β-yl)]-2-naphthamide* (**18a** for **19a**, **18b** for **19b** or **18c** for **19c**) (0.83 g, 2 mmol) was dissolved in anhydrous ethanol (150 mL) and catalytically hydrogenated (0.025 g of PtO_2_, 5 atm of H_2_, 24 h). The catalyst was filtered off and the filtrate was evaporated in vacuo to give crude **19a–c** as a white solid.

##### N-[8-(2-aminobenzyl)-8-azabicyclo[3.2.1]oct-3β-yl)]-2-naphthamide (**19a**)

Column chromatography chloroform: methanol (95: 5 v/v). Yield: 0.76 g (97.9%); m.p. 172.7–173.3 °C; ^1^H NMR (500 MHz, CDCl_3_): *δ* d 8.40 (C1”H); m 7.95–7.90 (C3”H, C8”H); m 7.89–7.85 (C4”H, C5”H); m 7.56–7.49 (C6”H, C7”H); td 7.03 (C4’H), ^3^*J* = 8.0, ^4^*J* = 1.5; dd 6.97 (C6’H), ^3^*J* = 7.5, ^4^*J* = 1.5; dd 6.76 (C3’H), ^3^*J* = 8.0, ^4^*J* = 1.0; td 6.63 (C5’H), ^3^*J* = 8.5, ^4^*J* = 1.0; bs 4.51 (NH_2_); m 4.40 (C3H(axial)); s 3.63 (C9H_2_); bs 3.25 (C1H, C5H); m 2.10 (C6H(E), C7H(E)); m 1.88–1.75 (C2H(E), C4H(E), C6H(A), C7H(A), C2H(A), C4H(A)); ^13^C NMR (125 MHz, CDCl_3_): *δ* 168.9 (C11); 147.9 (C2’); 135.9 (C4”a); 133.7 (C2”); 132.7 (C8”a); 130,8 (C6’); 129.8 (C8”); 129.0 (C4’); 128.9 (C5”); 128.7 (C1’); 128.5 (C4”); 128.4 (C7”); 127.4 (C6”); 125.0 (C3”); 124.7 (C1”); 118.9 (C5’); 117.3 (C3’); 59.2 (C1, C5); 55.7 (C9); 43.1 (C3); 38.2 (C2, C4); 27.0 (C6, C7); IR (KBr) cm^−1^: 3242 (NH_ar_), ν3237 (NH), 1634 (CO), ESI-HRMS *m*/*z* calcd for C_25_H_27_N_3_O (M + Na)^+^ 408.2052, found 408.2054.

##### N-[8-(3-aminobenzyl)-8-azabicyclo[3.2.1]oct-3β-yl)]-2-naphthamide (**19b**)

Column chromatography chloroform: methanol (85: 15 v/v). Yield: 0.75 g (97.3%); m.p. 165.4–166.6 °C; ^1^H NMR (500 MHz, CDCl_3_): *δ* d 8.43 (C1”H; m 7.97–7.91 (C3”h, C8”H); m 7.90–7.85 (C4”H, C5”H); m 7.53 (C6”H, C7”H); t 7.08 (C5’H), ^3^*J* = 8.0; t 6.9.0 (C2’H); dt 6.79 (C6’H), ^3^*J* = 8.0; m 6.71 (C4’H), ^3^*J* = 8.0, ^4^*J*_1_ = 2.5, ^4^*J*_2_ = 1.0; bs 4.56 (NH_2_); m 4.49 (C3H(axial)), s 3.77 (C9H_2_); s 3.56 (C1H, C5H); m 2.24 (C6H(E), C7H(E)); m 2.08 (C2H(E), C4H(E)); m 1.97 (C6H(A), C7H(A)); m 1.91 (C2H(A), C4H(A); ^13^C NMR (125 MHz, CDCl_3_): *δ* 170.0 (C11); 149.0 (C3’); 135.9 (C4”a); 133.7 (C2”); 132.4 (C8”a); 130.2 (C5’); 19.8 (C8”); 129.0 (C5”); 128.8 (C1’); 128.5 (C4”, C7”); 127.5 (C6”); 125.0 (C1”, C3”); 120.1 (C6’); 117.4 (C2’); 116.3 (C4’); 60.6 (C1, C5); 56.1 (C9); 41.9 (C3); 36.6 (C2, C4); 26.4 (C6, C7); IR (KBr) cm^−1^: 3309 (NH_ar_), ν1636 (CO); ESI-HRMS *m*/*z* calcd for C_25_H_27_N_3_O (M + Na)^+^ 408.2052, found 408.2049.

##### N-[8-(4-aminobenzyl)-8-azabicyclo[3.2.1]oct-3β-yl)]-2-naphthamide (**19c**)

Yield: 0.76 g (98.5%); m.p. 201.6–208.8 °C; ^1^H NMR (500 MHz, CDCl_3_): *δ* s 8.22 (C1”H); m 7.86–7.81 (C3”H, C4”H, C8”H); dd 7.79 (C5”H), ^3^*J* = 8.5,^4^*J* = 1.5; m 7.53 (C6”H, C7”H); d 7.16 (C2’H, C6’H), ^3^*J* = 8.5; d 6.65 (C3’H, C5’H), ^3^*J* = 8.5; d 6.12 (NH), ^3^*J* = 8.5; m 4.42 (C3H(axial)); bs 3.61 (NH_2_); s 3.45 (C9H_2_); bs 3.28 (C1H, C5H); m 2.07 (C6H(E), C7H(E)); m 1.95 (C2H(E), C4H(E)); pq 1.78 (C6H(A), C7H(A)); td 1.66 (C2H(A), C4H(A)), ^3^*J*_A–A_ = 12.0, ^3^*J*_A–E_ = 2.0; ^13^C NMR (125 MHz, CDCl_3_): *δ* 166.7 (C11); 145.3 (C4’); 134.6 (C4”a); 132.6 (C2”); 132.1 (C8”a); 129.8 (C2’, C6’); 129.6 (C1’); 128.9 (C8”); 128.4 (C5”); 127.7 (C4”); 127.5 (C7”); 127.2 (C6”); 126.7 (C3”); 123.6 (C1”); 115.0 (C3’, C5’); 58.6 (C1, C5); 55.8 (C9); 42.1 (C3); 38.7 (C2, C4); 26.4 (C6, C7); IR (KBr) cm^−1^: 3330 (NH_ar_), 1638 (CO), ESI-HRMS *m*/*z* calcd for C_25_H_27_N_3_O (M + H)^+^ 386.2226, found 386.2232.

### HPLC analysis

Dionex system was used. The system consisted of a quaternary pump P580, a UVD detector 340 S, a column thermostat YetStream II Plus (WO Industrial Electronics), all controlled with Chromeleon software (version 6.01). Sample injection was performed through Rheodyne injector valve with a 20 µl sample loop. Chromatographic separations were carried out using the NUCLEODUR C18 Gravity column (Machery-Nagel), 150 × 4.6 mm, 5 µm and guard column NUCLEODUR C18 Gravity 5 µm. Mobile phases consisted of a mixture of 6 mM octane-1-sulphonic acid sodium salt and MeOH (55: 45) adjusted the pH to 3 with acetic acid. The flow rate of the mobile phase was 0.8 ml/min. The temperature in the column was maintained at 30 °C. Thanks to the diode array detector, it was possible to record UV spectra of analysed compounds with absorbance maximum at c.a. 236 nm. Detection was carried out at *λ* = 236 nm.

### Biological tests

#### Radioligand binding assay

All compounds were tested for their affinities for 5-HT_1A_, 5-HT_2A_, and D_2_ receptors according to previously described procedures (Stefanowicz et al. 2016).

#### In vivo studies

##### Animals

The experiments were performed on male mice (22–26 g, Albino Swiss or CD-1). All animals were kept in an environmentally controlled rooms (ambient temperature 21 ± 2 °C; relative humidity 50–60%; 12:12 light–dark cycle, lights on at 8:00) and filtered water were freely available. All the experimental procedures were approved by the I Local Ethics Commission at the Jagiellonian University in Krakow. All the experiments were conducted in the light phase between 09:00 and 14:00 h. Each experimental group consisted of 6–10 animals/dose, and the animals were used only once in each test.

#### Spontaneous locomotor activity

The locomotor activity was recorded with an Opto M3 multi-channel activity monitor (MultiDevice Software v.1.3, Columbus Instruments). The investigated compounds or vehicle were administered intraperitoneally (i.p.) 60 min before the test running. The mice were individually placed in plastic cages (22 × 12 × 13 cm) for 30 min habituation period, and then the crossings of each channel (ambulation) were measured every 5 for 60 min (in CD-1 mice) and during 1-min or 3–6 min test session for Albino Swiss mice. The cages were cleaned up with 70% ethanol after each mouse.

#### MK-801-induced hyperactivity

MK-801-induced hyperactivity in mice was recorded according to the method described above. The investigated compounds or vehicle were administered i.p. 30 min., while MK-801 0.2 mg/kg i.p. 15 min before the test running.

#### Amphetamine-induced hyperactivity

d-Amphetamine-induced hyperactivity in mice was recorded according to the method described above. The investigated compounds or vehicle were administered i.p., while amphetamine 2.5 mg/kg subcutaneously (s.c.) 30 min before the test running.

#### Forced swim test in mice

The experiment was carried out according to the method of Porsolt et al. (1979). Mice (Swiss Albino) were individually placed in a glass cylinder (25 cm high; 10 cm in diameter) containing 10 cm of water maintained at 23–25 °C, and were left there for 6 min. A mouse was regarded as immobile when it remained floating on the water, making only small movements to keep its head above it. The total duration of immobility was recorded during the last 4 min of a 6-min test session.

#### Four-plate test in mice

Test was performed on male Swiss Albino mice. A single mouse was placed gently onto the plate, and each animal was allowed to explore for 15 s. Afterwards, each time a mouse passed from one plate to another, the experimenter electrified the whole floor for 0.5 s (current 0.8 mA), which evoked a visible flight reaction of the animal. If the animal continued running, it received no new shock for the following 3 s. The number of punished crossings was counted for 60 s.

### Statistical analysis

The data are presented as the mean ± S.E.M.The obtained data were analysed by one-way analysis of variance (ANOVA) followed by Bonferroni’s post-hoc test. *p* < 0.05 were considered statistically significant.

## Results and discussion

### Chemistry

Final compounds **12a–w** and **19a–c** were obtained via a multi-step synthesis according to Scheme 1 and Scheme 2. The starting compounds (**1**–**9**, **13**–**17**) were synthesised according to procedures described in our previous paper (Słowiński et al. 2011; Stefanowicz et al. 2016). The 8-benzyl-8-azabicyclo[3.2.1]oct-3β-yl-amine (**7**) required for synthesis of the final compounds were obtained in several steps. The starting 8-benzyl-8-azabicyclo[3.2.1]octan-3-one (**5**) were synthesised from the benzyl amine via a modified Robinson condensation (Dostert et al. 1984). The obtained ketone (**5**) was subsequently converted to an oxime (**6**), which was then subjected to a stereoselective reduction with sodium in butanol to give the equatorial (*β*) 8-benzyl-8-azabicyclo[3.2.1]oct-3*β*-yl-amine (**7**). The above reactions were carried out using methods described in the literature (Dostert et al. 1984). The 8-benzyl-8-azabicyclo[3.2.1]oct-3β-yl-acetamide (**8**) were synthesised by the acylation of compound **7** by treatment with acetyl chloride in the presence of triethylamine as a base and dichloromethane as a solvent (Dostert et al. 1984). The N-[8-aryl-8-azabicyclo[3.2.1]oct-3β-yl]acetamide derivatives (**10a–f)** were obtained from the known intermediate 8-azabicyclo[3.2.1]oct-3β-yl-acetamide hydrochloride **9**, via debenzylation to amide **8**, which was then alkylated with the appropriate benzylmethyl chloride or pyridinemethyl chlorides using the Finkelstein protection of KI (Scheme 1). Compounds **10a–f** have not been described before in the literature.

The next stage was acid catalysed hydrolysis of the amide bond of **10a–f** derivatives, giving 8-aryl-8-azabicyclo[3.2.1]oct-3β-yl-amine derivatives (**11a–f**). Due to the high process yield and purity of the crude products, compounds **11a–f** were used in subsequent reactions without further purification. The mixed anhydride method was used in order to obtain the final planned *β*-quinolineamide derivatives (**12a–w**).

All except one of the reported synthesis methods for the final compounds proved to be stereospecific. However, compound **12u** was obtained as a mixture of isomers (see Scheme 1). This observation is of particular interest in view of earlier our research results of group 3β-acylamine derivatives of tropane. The ratio of **12u** isomers in the mixture was confirmed by ^1^H NMR and HPLC spectral analysis as described in section Conformational analysis.

The synthesis of N-[8-(aminobenzyl)-8-azabicyclo[3.2.1]oct-3β-yl)]-2-naphthamide derivatives (**19a–c**) were accomplished according to Scheme 2. The N-(8-azabicyclo[3.2.1]oct-3*β*-yl)-2-naphthamide hydrochloride (**17**) required for synthesis of the final compounds were obtained from the known intermediate *N*-(8-methyl-8-azabicyclo[3.2.1]oct-3*β*-yl)-2-naphthamide (**16**) via demethylation with Olofson’s reagent. The above reactions were carried out using methods described in our previous paper (Stefanowicz et al. 2016). Next, **17** was alkylated with the appropriate nitrobenzyl chlorides to give the corresponding N-[8-(nitrobenzyl)-8-azabicyclo[3.2.1]oct-3β-yl)]-2-naphthamides derivatives (**18a–c**). In the next stage, appropriate **18a–c** derivatives were subjected to catalytic hydrogenation of the nitro group in the presence of PtO_2_ to give final compounds **19a–c**.

The structures of all novel intermediates and final compounds were confirmed by IR, ^1^H NMR and ^13^C NMR spectroscopy and ESI-HRMS spectrometry. Detailed characterisation data are provided in Material and methods section. For in vivo and in vitro investigations, free bases were converted into the corresponding water-soluble salts.

### Conformational analysis

**Fig. 4:**
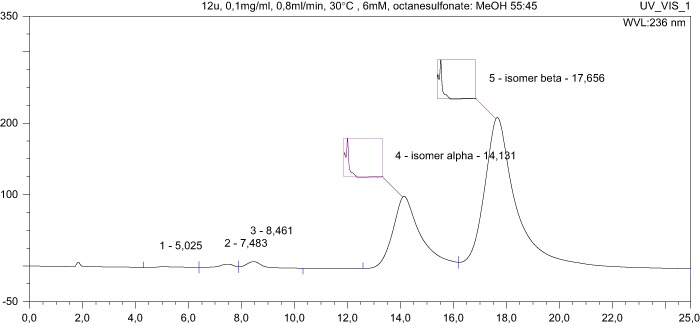
Chromatogram of **12u** (mixture of stereoisomers α and β)

**Fig. 5:**
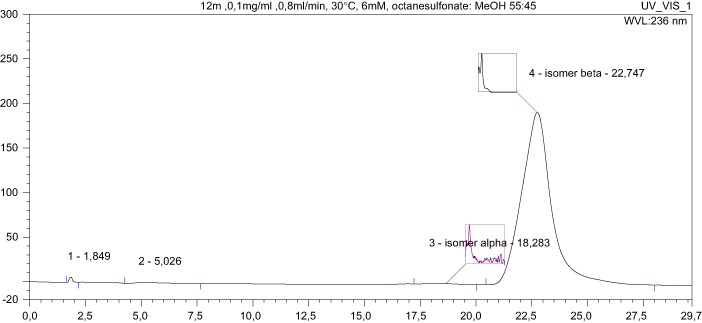
Chromatogram of **12m** (mixture of stereoisomers α and β)

The ^1^H and ^13^C NMR spectra of the samples **12a**–**w** confirm the assumed structures (see Material and methods section). The signal of the C3H proton (in proton spectra) is particularly interesting as it has the form of a 12-line or 14-line multiplet. In order to account for this splitting pattern we need to assume that the C3H proton is axial. For the 12-line presentation, we can assume that the multiplet is formed of 3 overlapping quartets, this corresponding to an initial split into a triplet by axial C2H and C4H protons followed by the triplet constituents splitting into quartets as a result of coupling with the three protons of C2H and C4H (equatorial) and NH. We assume that the NH proton coupling constant is the same as (or very similar to) the constants of coupling to the equatorial protons of C2H and C4H. This assumption cannot hold for the 14-line presentation and it can be assumed in this latter case that the signal from the C3H proton is split into a triplet by coupling with the axial C2H and C4H protons, followed by a split of the triplet components into doublets by the NH proton and, finally, followed by the components of the three doublets being split by equatorial C2H and C4H protons. As a result there are 18 theoretical lines, but partial signal overlap simplifies the multiplet to 14 lines, confirming our assumption that the C3H proton is axial, but also leading to the conclusion that the spatial position of the equatorial -NH-CO-R substituent is different in the different compounds analysed (Figs. 4 and 5).

An unexpected effect is seen in the spectra of sample **12u**, where there is a marked increase in the number of signals in the ^13^C and ^1^H NMR spectra, suggesting the presence of a mixture of compounds. Fortunately, the finding of distinct multiplets of the C3H proton (in the proton spectrum) allows the conclusion that there is a mixture of two isomers: one (β) with an axial multiplet of the C3H proton (12 lines) and one (α) with an equatorial multiplet of the C3H proton (4 lines). Apparently, the equatorial C3H proton couples with three protons, namely, the axial C2H and C4H, and NH to produce a pseudoquartet. This requires making the assumption that the coupling constant for C2H and C4H equals 0. Then (according to the Karplus curve), the C3H coupling plane forms an angle of ~90^o^ with the coupling planes of C2H and C4H. Integrals (in ^1^H NMR spectra) can be used to calculate that the molar ratio of the β form to the α form is 2:1. This conclusion is corroborated by a good fit of the chemical shifts in ^1^H and ^13^C NMR spectra with the spectra of similar structures.

HPLC studies were conducted following determination of NMR spectra, which revealed that **12u** is a mixture of stereoisomers. The synthesis was repeated twice, with HPLC analysis producing very similar results to NMR spectral analysis.

The UV spectra (see supplementary material) are identical and characterised by the same absorbance maximums; i.e., at wavelengths equal to their absorbance maximum, both isomers are detected at the same maximum sensitivity. In this situation, the mass ratio of the isomers can be determined by comparing peak areas.

The peak area for **12u** α, at *t*_R_ = 14.131 min., is 125.1019 mAU × min., compared to 300.2409 mAU × min for **12u** β, at *t*_R_ = 17.656 min., producing an α:β ratio of 1:2.4, corresponding to an approximately 29.4% admixture of the **12u** α isomer.

To check for stereochemical purity, the analysis was repeated for the remaining compounds. This paper contains the results for the compound **12m**. The area of the **12m** α peak, at *t*_R_ = 18.283 min., is 2.0553 mAU × min., and the area of the **12m** β peak, at *t*_R_ = 22.747 min., is 300.1300 mAU × min., producing an α:β ratio of 1:146, which corresponds to an admixture of the **12m** α isomer of approximately 0.7%.

In summary, all target compounds are equatorial isomers (3β), except for the derivative **12u**. To our surprise, the admixture of an axial isomer (3α) was significant (29.4% by HPLC) only in this case. The same results were seen with the re-synthesised compound. We are unable to account for this isomerisation, the less so as the analogues **12t**, **12v**, and **12w** obtained from the same stereochemically pure substrate **10f** (NMR) are stereochemically pure equatorial isomers. Work to explain this is under way.

### Biological evaluation

#### Radioligand binding assay for D_2_, 5-HT_1A_, and 5-HT_2A_ receptors

As mentioned in the Introduction, ligands with simultaneous affinity for D_2_, 5-HT_1A_, and 5-HT_2A_ receptors seem to be promising compounds for the pharmacotherapy of schizophrenia. In our previous paper, we described the synthesis and biological evaluation of compounds with very good double binding to D_2_ and 5-HT_2A_ receptors; the most potent are shown in Fig. 1. Thus, in the subsequent phase of experimentation, we focused our attention on evaluating the impact of lead structure modification on 5-HT_1A_ receptor affinity. Therefore, the compounds synthesised within the present project included structural analogues of 3β-acylamine derivatives of tropane with the introduction of a methyl substituent in the benzyl ring and a quinoline moiety. These modifications were designed as a result of previous research, aiming to develop new ligands with enhanced 5-HT_1A_ binding activity in the investigated group of tropane derivatives.

Compounds **12a–w** and **19a–c** were tested for their in vitro affinity for the D_2_, 5-HT_1A_, and 5-HT_2A_ receptors using a radioligand binding assay. Competition binding studies were performed according to a previously described procedure in rat brain tissues (Stefanowicz et al. 2016). The results are presented in Table 1.

**Table 1:**
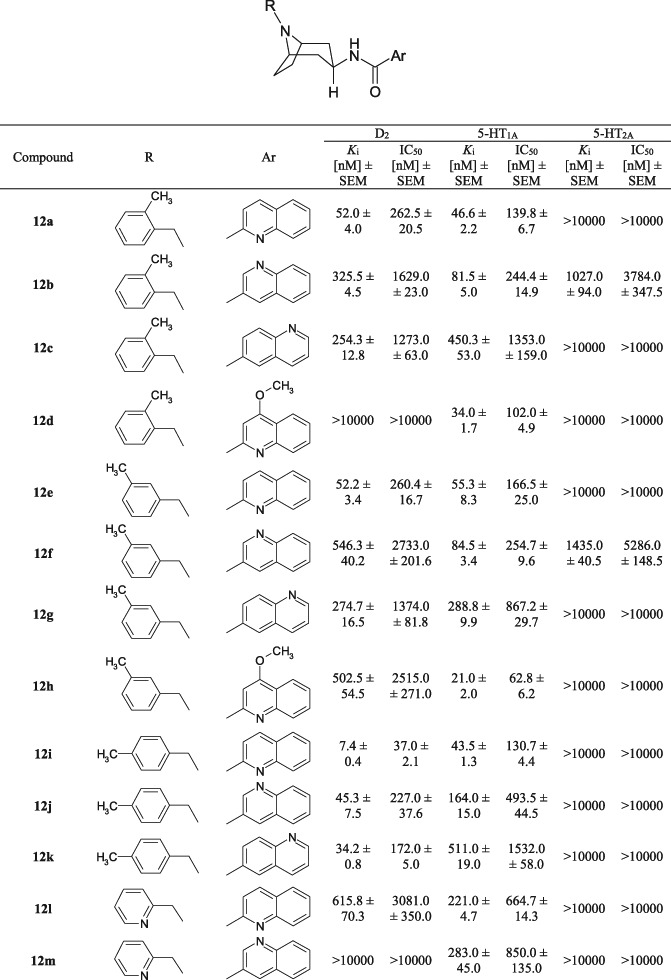

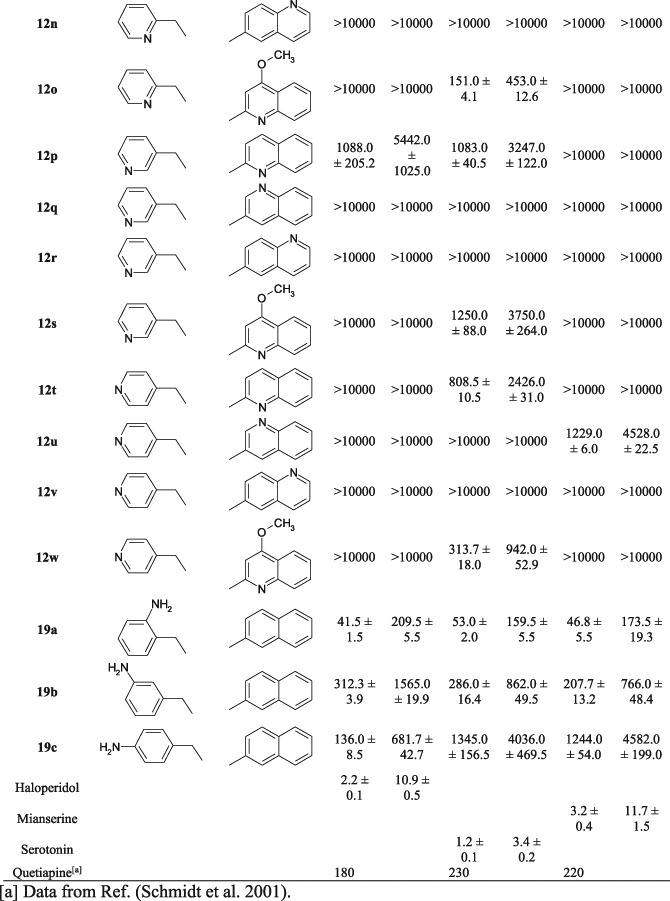
Binding affinities for dopamine D_2_ and serotonin 5-HT_1A_/5-HT_2A_ receptors

First, the impact of structure modifications to the quinoline derivatives (**12a–w**) on D_2_ affinity were examined. The influence of the quinoline moiety and its derivatives were analysed. The nitrogen position in the quinolinyl fragment impacted on the affinity for D_2_. We observed the same rank order: 2-quinolinyl > 6-quinolinyl > 3-quinolinyl in the case of all ligands. Therefore, introduction of 2-quinoline fragment in ligands was found to be favourable for D_2_ binding, with the highest affinity seen for compound **12i**. At the same time, the 4-methoxy-quinoline analogues had in general the lowest affinity for this receptor.

Next, by comparing the influence the location of the methyl substitution in the benzyl ring, it was confirmed that *p*-substituted ligands (**12i, 12j, 12k**) were considerably more potent than their *m-*substituted or *o-*substituted analogues, with compound **12i** (*p-*CH_3_) displaying a D_2_ *K*_i_ = 7.4 nM. This is in accordance with our previous results (Stefanowicz et al. 2016).

The substitution of a 2-piridylmethyl, 3-piridylmethyl, or 4-piridylmethyl at the in N8 position resulted in a significant loss of activity at the D_2_ receptor.

Analysing the *K*_i_ values for 5-HT_1A_ receptors, we found that the introduction of quinoline and its derivatives was beneficial in terms of 5-HT_1A_ receptor binding affinity. Furthermore, the presence of the 4-methoxy-2-quinoline moiety notably ameliorated the affinity for the 5-HT_1A_ receptor. This enhancing effect was greatest for compound **12h** *K*_i_ = 21.0 nM. The nitrogen position in the quinolinyl fragment also affected binding to 5-HT_1A_ receptors; these could be ranked in order of their increasing influence as follows: 2-quinolinyl > 3-quinolinyl > 6-quinolinyl.

After analysis of the results obtained in the radioligand binding assay, it was concluded that very high affinity for the 5-HT_1A_ receptor was demonstrated by the following ligands: **12a** (*K*_i_ = 46.6 nM), **12d** (*K*_i_ = 34.0 nM), **12h** (*K*_i_ = 21.0 nM), **12i** (*K*_i_ = 43.5 nM). Taking into consideration the impact of *o*-methyl, *m*-methyl, or *p*-methyl substituents located in the benzyl ring on affinity for the 5-HT_1A_ receptor, it can be stated that the substituents generally did not have a significant effect on affinity compared to the non-substituted analogues. The *o*-methyl, *m*-methyl, or *p*-methyl benzyl with 2-quinolinyl moiety derivatives showed good binding affinity, especially **12a** (*K*_i_ = 46.6 nM), **12e** (*K*_i_ = 55.3 nM), and **12i** (*K*_i_ = 43.5 nM), compared to the corresponding non-substituted analogue N-(8-benzyl-8-azabicyclo[3.2.1]oct-3b-yl)-quinoline-2-carboxamide (*K*_i_ = 62.7 nM). As well, the 5-HT_1A_ receptor affinity of the substituted 4-methoxy quinolinyl analogues **12d** (*K*_i_ = 34.0 nM) and **12h** (*K*_i_ = 21.0 nM) can be compared to the corresponding non-substituted analogue N-(8-benzyl-8-azabicyclo[3.2.1]oct-3b-yl)-4-methoxyquinoline-2-carboxamide (*K*_i_ = 30.5 nM).

In contrast 2-pyridine, 3-pyridine, or 4-pyridine derivatives (**12l**-**w**) displayed dramatically lower binding affinity for 5-HT_1A_ receptors than their benzene substituted ligands. The highest affinity was observed for the 2-pyridine analogue **12o** (*K*_i_ = 151.0 nM).

It is worth mentioning that the replacement of the naphthyl ring with heterocyclic analogues led to the complete loss of 5-HT_2A_ receptor affinity in the investigated group of ligands. Thus, the presence of a naphthyl moiety is crucial for obtaining ligands in this series with triple binding activity for the D_2_, 5-HT_1A_, and 5-HT_2A_ receptors.

The introduction of an additional nitrogen atom into the molecule as an amine group in the phenyl ring (Fig. 2) in derivatives **19a-c** resulted in a marked increase in affinity for all receptors under study. The resulting compounds showed the highest binding affinity for the D_2_, 5-HT_1A_, and 5-HT_2A_ receptors of all derivatives described in this paper. In this respect, the compound **19a** (*K*_i_[nM] = D_2_ = 41.5; 5-HT_1A_ = 53.0; 5-HT_2A_ = 46.8) appears to hold the greatest promise. The affinity of the derivative **19a** described above and its analogues **19b** and **19c** was markedly influenced by the position of the -NH_2_ moiety in the benzyl system, where the *o*-isomer was the most active one and the *p*-isomer was the least active. Of note, unlike the other new structures, these three compounds are naphthalene derivatives. This again seems to lead to the conclusion that the presence of a naphthalene system in these compounds is more beneficial than the presence of a quinoline system in terms of producing a derivative with triple binding affinity for the D_2_, 5-HT_1A_, and 5-HT_2A_ receptors. The salts of compounds **19a**–**c** were also characterised by the best solubility in water among all the derivatives analysed.

In summary, the introduction of an additional nitrogen atom into the naphthalene or phenyl ring had an overall adverse effect on the binding affinities of the new compounds compared to the lead structure (**compound A**) and its derivatives described in our previous publication. This modification had the greatest negative effect on affinity for the 5-HT_2A_ receptor. Of note, the 2-quinoline derivatives **12e** and **12i** demonstrated very good binding affinity for the D_2_ and 5-HT_1A_ receptors, being superior in this respect to the 3- and 6-quinoline analogues.

The introduction of a pyridine ring into the molecule had an adverse effect on binding affinity, while the introduction of an amine group as a substituent in the phenyl ring produced very active compounds. Compound **19a** is exceptional among the analysed structures as it demonstrates comparable affinities for all three receptor types (D_2_, 5-HT_1A_, and 5-HT_2A_), resulting in a very quetiapine-like receptor profile, but with binding affinities 4-fold or 5-fold higher than those of quetiapine.

#### In vivo studies

##### General

Experiments were carried out on Albino Swiss or CD-1 male mice weighing 22–26 g kept in colony cages in standard laboratory conditions. Experimental groups were chosen randomly and each animal was used only once. The compounds studied were suspended in a 1% solution of Tween 80 (Sigma, St. Louis, MO, USA) and injected intraperitoneally in a volume of 10 ml/kg.

#### Antipsychotic-like activity

To study the potential antipsychotic activity of selected compounds the d-amphetamine- and MK-801-induced hyperlocomotor activity test in mice were carried out. Compounds **12i** (5 mg/kg and 10 mg/kg i.p.), **19a** (5 mg/kg and 10 mg/kg i.p.) significantly reduced MK-801-induced hyperlocomotor activity (Table 2). Compound **12e** administered at a dose of 5 mg/kg showed a tendency to decrease the MK-801-induced hyperlocomotor activity but the results did not reach a statistically significant level (Table 2). In d-amphetamine-induced hyperlocomotor activity test, compounds **12i** and **12e** (at doses of 5 and 10 mg/kg i.p.) significant decreased locomotor hyperactivity in the range of 60–86% vs. respective d-amphetamine group (Table 3). The compound **19a** was active in this test only at a dose of 10 mg/kg i.p. (Table 3).

**Table 2:** Effects of **12e**, **12i**, and **19a** on the MK-801-induced hyperlocomotor activity in CD-1 mice

Treatment	Dose (mg/kg)	Number of crossings/60 min Mean ± SEM
Control	0	3243.3 ± 519.8
MK-801	0.2	6329.9 ± 1012.5; *p* < 0.05 vs. contr
**12e** + MK-801	0.625 + 0.2	4375.1 ± 583.4; ns, ns
	1.25 + 0.2	4019.3 ± 341.3; ns, ns
	2.5 + 0.2	5440.1 ± 937.3 ns, ns
	5 + 0.2	3580.7 ± 384.9; ns, ns F(5,48) = 2.8885; *p* < 0.05
Control	0	2362.7 ± 400.8
MK-801	0.2	4708.1 ± 522.1; *p* < 0.01 vs. contr
**12i** + MK-801	5 + 0.2	2432.8 ± 526.6; ns vs. contr *p* < 0.01 vs. MK
	10 + 0.2	805.4 ± 147.2 ns vs. contr *p* < 0.0001 vs. MK F(3,30) = 14.206; *p* < 0.0001
Control	0	1629.7 ± 235.6
MK-801	0.2	6247.1 ± 1063.8; *p* < 0.01 vs. contr
**19a** + MK-801	1.25 + 0.2	4646.4 ± 675.6; ns, ns
	2.5 + 0.2	3832.0 ± 991.3; ns, ns F(3,33) = 5.2088; *p* < 0.01
Control	0	2068.3 ± 387.3
MK-801	0.2	8757.9 ± 1378; *p* < 0.00001 vs. contr
**19a** + MK-801	5 + 0.2	2915.4 ± 580.2; *p* < 0.001 vs. MK; ns vs. contr F(2,27) = 16.679; *p* < 0.0001
Control	0	2362.7 ± 400.8
MK-801	0.2	4708.1 ± 522.1; *p* < 0.01 vs. contr
**19a** + MK-801	10 + 0.2	808.7 ± 66.3; *p* < 0.0001 vs. Mk; *p* < 0.05 vs. contr F(2,20) = 23.813; *p* < 0.001

The investigated compounds were injected i.p. 30 min, while MK-801 15 min. before the test. Values represent the mean ± SEM during 60-min test session compared to the respective group (one-way ANOVA is followed by the Bonferroni’s post hoc test), *N* = 8–10, NS–non-significant

**Table 3:** Effects of **12e**, **12i**, and **19a** on the d-amphetamine-induced hyperlocomotor activity in CD-1 mice

Treatment	Dose (mg/kg)	Number of crossings/60 min Mean ± SEM
Control	0	2011.7 ± 358.8
d-amphetamine	2.5	6553.1 ± 1214.5; *p* < 0.001 vs. contr
**12e** + d-amphetamine	2.5 + 2.5	2993.0 ± 301.0; *p* < 0.01 vs. amph.; ns vs. contr
	5 + 2.5	2651.0 ± 512.6; *p* < 0.01 vs. amph.; ns vs. contr
	10 + 2.5	909.9 ± 11.3; *p* < 0.0001 vs. amph.; ns vs. contr F(4,42) = 10.584; *p* < 0.0001
Control	0	2664.2 ± 540.9
d-amphetamine	2.5	6819.9 ± 1534.7; *p* < 0.05 vs. contr
**12i** + d-amphetamine	5 + 2.5	1771.3 ± 470.4; *p* < 0.01 vs. amph.; ns vs. contr
	10 + 2.5	2046.2 ± 63.1; *p* < 0.01 vs. amph.; ns vs. contr F(3,34) = 6.5110; *p* < 0.01
Control	0	2664.2 ± 540.9
d-amphetamine	2.5	6819.9 ± 1534.7; *p* < 0.05 vs. contr
**19a** + d-amphetamine	5 + 2.5	4328.6 ± 1146.8
	10 + 2.5	950.0 ± 411.7; *p* < 0.01 vs. amph.; ns vs. contr F(3,35) = 5.6910; *p* < 0.01

The investigated compounds were injected i.p., while d-amphetamine s.c., 30 min. before the test. Values represent the mean ± SEM during 60-min test session compared to the respective group (one-way ANOVA is followed by the Bonferroni’s post hoc test), N = 8–10, NS–non-significant

The compounds **19a** (10 mg/kg) and **12i** (10 mg/kg) significantly decreased spontaneous locomotor activity about 70% since the positive effects observed in hyperlocomotor activity tests may not be specific (Table 4). The compound **12e** at the doses used in hyperlocomotor activity tests did not change the spontaneous locomotor activity in mice, thus its antipsychotic-like effect appeared to be specific (Table 4).

**Table 4:** Effects of **12e**, **12i**, and **19a** on the spontaneous locomotor activity in CD-1 mice

Treatment	Dose (mg/kg)	Number of crossings/60 min Mean ± SEM
Control	0	3352.9 ± 966.4
**12e**	10	1034 ± 255.9 *p* < 0.05 F(1,18) = 5.4398; *p* < 0.05
Control	0	1280.2 ± 222.2
**12i**	1.25	1573.6 ± 149.3
	2.5	2676.0 ± 336.7; *p* < 0.01
	5	1135.8 ± 184.3 F(3,34) = 8.8733; *p* < 0.001
Control	0	2068.3 ± 387.3
**19a**	1,25	2467,9 ± 539.3
	2,5	1634.4 ± 511.9 F(2,27) = 0.7417; NS
Control	0	1280.2 ± 222.2
**19a**	5	1480.4 ± 302.3
	10	491.6 ± 126.5 F(2,26) = 5.3557; *p* < 0.05

The compounds were injected i.p. 30 min, before the test. Values represent the mean ± SEM during 60-min test session compared to the respective vehicle group (one-way ANOVA is followed by the Bonferroni’s post hoc test), *N* = 8–10, NS–non-significant

#### Antidepressant-like activity

The potential antidepressant activity of selected compounds in vivo was investigated using the forced swim test in mice. In this test only compound **19a** (5 mg/kg i.p.) decreased immobility time about 43% vs. respective control group, showing significant antidepressant-like activity (Table 5).

**Table 5:** Effects of **12d**, **12e**, **12h**, **12i**, and **19a** in the forced swim test in Albino Swiss mice

Treatment	Dose (mg/kg)	Immobility time (s) Mean ± SEM
Control	0	142.10 ± 8.9
**12d**	1.25	144.22 ± 14.5
	2.5	113.44 ± 14.9
	5	150.8 ± 11.4 F(3,32) = 5.5680; NS
Control	0	183.56 ± 12.4
**12e**	2.5	172.14 ± 11.6
	5	15.44 ± 16.8
	10	183.33 ± 12.3 F(3,30) = 1.2074; NS
Control	0	142.10 ± 8.9
**12h**	0.625	163.29 ± 10.7
	1.25	134.78 ± 12.9
	2.5	185.00 ± 15.0 F(3,30) = 3.5680; *p* < 0.05
Control	0	157.33 ± 16.1
**12i**	1.25	132.00 ± 2.93
	2.5	149.89 ± 12.7
	5	219.67 ± 1.43; *p* < 0.05 F(3,26) = 7.5750; *p* < 0.001
Control	0	168.50 ± 5.9
**19a**	1.25	120.00 ± 16.6
	2.5	128.89 ± 12.3
	5	96.56 ± 16.2; *p* < 0,01 F(3,31) = 4.5279; *p* < 0.01
Control	–	162.71 ± 6.8
Imipramine	5	170.42 ± 10.9
	10	119.63 ± 13,0; *p* < 0.05
	20	77.81 ± 12.2; *p* < 0.001 F(3,36) = 16.7570; *p* < 0.0001

The compounds were injected i.p. 30 min. before the test. Values represent the mean ± SEM during last 4-min test session compared to the respective vehicle group (one-way ANOVA is followed by the Bonferroni’s post hoc test), *N* = 6–9, NS–non-significant

#### Anxiolytic-like activity

The potential anxiolytic activity of selected compounds in vivo was investigated using the four-plate test in mice. In this test only compound **12h** (1.25 and 2.5 mg/kg i.p.) increased punished crossings in a range of 60% vs. respective control group, showing significant anxiolytic-like activity (Table 6).

**Table 6:** Effects of **12d**, **12e**, **12h**, **12i**, and **19a** in the four-plate test in Albino Swiss mice

Treatment	Dose (mg/kg)	Number of punished crossings /1 min Mean ± SEM
Control	0	3.3 ± 0.2
**12d**	2.5	3.3 ± 0.3
	5	4.8 ± 0.3
	10	4.7 ± 0.8 F(3,36) = 3.1652; *p* < 0.05
Control	0	3.3 ± 0.2
**12e**	2.5	3.5 ± 0.3
	5	2.9 ± 0.3
	10	3.8 ± 0.5 F(3,36) = 1.3827; NS
Control	0	3.3 ± 0,2
**12h**	1.25	5.3 ± 0.3; *p* < 0.001
	2.5	5.5 ± 0.5; *p* < 0.001
	5	2.9 ± 0.3 F(3,36) = 17.202; *p* < 0.00001
Control	0	3.6 ± 0.5
**12i**	2.5	3.1 ± 0.3
	5	2.3 ± 0.4
	10	1.7 ± 0.4; *p* < 0.05 F(3,35) = 4.2954; *p* < 0.05
Control	0	2.8 ± 0.5
**19a**	2.5	3.2 ± 0.5
	5	1.1 ± 0.2
	10	3.2 ± 0.5 F(3,33) = 5.6670; *p* < 0.01
Control	–	4.2 ± 0.4
Diazepam	1.25	5.8 ± 0.3; *p* < 0.01
	2.5	6.4 ± 0.5; *p* < 0.01
	5	6.6 ± 0.4; *p* < 0.05 F(3,36) = 6.455; *p* < 0.01

The investigated compounds were injected i.p. 30 min., while diazepam 60 min. before the test. Values represent the mean ± SEM during 1-min test session compared to the respective vehicle group (one-way ANOVA is followed by the Bonferroni’s post hoc test), *N* = 8–10, NS–non-significant

Active doses of the investigated compounds had no influence on the spontaneous locomotor activity measured during the time equal to the observation period in the forced swim and the four-plate tests (i.e., from 2–6 min and 1 min 15 s, respectively) (data not shown) thus observed antidepressant-like and/or anxiolytic-like activity of these compounds seems to be specific.

## Conclusion

We have described here a series of 26 compounds representing new derivatives of 3β-aminotropane and being analogues of a previously identified compound A, which shows high activity at the D_2_, 5-HT_1A_, and 5-HT_2A_ receptors. Modifications involved the introduction of an additional nitrogen atom, producing quinoline, isoquinoline or pyridine derivatives or derivatives with an amine group as a substituent in the phenyl ring (Fig. 2). Structure-activity relationship studies revealed that these modifications adversely affected the binding affinity of these compounds for the three types of receptors, except for the derivatives **12e** and **12i**, which demonstrated high binding affinity for the D_2_ and 5-HT_1A_ receptors, and the compound **19a**, which showed comparable binding affinities for all three receptor types (D_2_, 5-HT_1A_, and 5-HT_2A_), giving it a very quetiapine-like receptor profile, but with 4-fold or 5-fold higher binding affinities than that antipsychotic drug.

Studies of behavioural activity of selected compounds (**12d**, **12e**, **12h**, **12i**, and **19a**) showed that the compounds **12i** and **19a** exerted a specific antipsychotic-like effect in d-amphetamine-induced and MK-801-induced hyperlocomotor activity test in mice. Specific antidepressant-like activity (the forced swim test) was displayed only by the compound **19a** and a specific anxiolytic-like effect was produced only by **12h** (Figs. 3–5).

The beneficial and more comprehensive activity profile of the compound **19a** encourages further rational search for new antipsychotics with an affective component in this structural class.

## Electronic supplementary material

Supplementary Information
