# Supplementary material for: Synthesis and biological investigations of 3β-aminotropane arylamide derivatives with atypical antipsychotic profile
Source: Med Chem Res. 2018 Jun 22;27(8):1906–28. doi: 10.1007/s00044-018-2203-z (PMC6061170; doi:10.1007/s00044-018-2203-z)

**Medicinal Chemistry Research**

**Supplementary material**

**Synthesis and biological investigations of 3β-aminotropane arylamide derivatives with atypical antipsychotic profile.**

**Jacek Stefanowicz^1^ ‧ Tomasz Słowiński^1^ ‧ Martyna Z. Wróbel^1^ ‧ Grzegorz Ślifirski^1^ ‧ Maciej Dawidowski^1^ ‧ Zdzisława Stefanowicz^2^ ‧ Magdalena Jastrzębska-Więsek^3^ ‧ Anna Partyka^3^ ‧ Anna Wesołowska^3^ ‧ Jadwiga Turło^1^**

🖂 Martyna Z. Wróbel

[martyna.wrobel@wum.edu.pl](mailto:martyna.wrobel@wum.edu.pl)

[martynawrobel.wum@gmail.com](mailto:martynawrobel.wum@gmail.com)

^1^ Department of Drug Technology and Pharmaceutical Biotechnology, Faculty of Pharmacy, Medical University of Warsaw, 1 Banacha Street, 02-097 Warsaw, Poland

^2^ Department of Inorganic and Analytical Chemistry, Faculty of Pharmacy, Medical University of Warsaw, 1 Banacha Street, 02-097 Warsaw, Poland

^3^ Department of Clinical Pharmacy, Jagiellonian University Medical College, 9 Medyczna Street, 30-688 Cracow, Poland

**Contents**

**1. Synthesis of:**

1.1 8-phenylmethyl-8-azabicyclo[3.2.1]octan-3-one oxime (**6**) and 8-phenylmethyl-8-azabicyclo[3.2.1]oct-3b-ylamine (**7**)

1.2 8-phenylmethyl-8-azabicyclo[3.2.1]oct-3β-ylacetamide (**8**) and 8-azabicyclo[3.2.1]oct-3β-ylacetamide hydrochloride (**9**)

1.3 8-methyl-8-azabicyclo[3.2.1]octan-3-one oxime (**14**)

1.4 8-methyl-8-azabicyclo[3.2.1]oct-3b-ylamine (**15**)

1.5 (8-Methyl-8-azabicyclo[3.2.1]oct-3β-yl)-2-naphthamide (**16**)

1.6 N-(8-azabicyclo[3.2.1]oct-3β-yl)-2-naphthamide hydrochloride (1**7**)

1.7 References

**2. UV spectra of the stereoisomers α and β, compounds 12u and 12m**

**3. ^1^H NMR spectra of final compounds 12a-w and 19a-c**

- 1. Synthesis of 8-phenylmethyl-8-azabicyclo[3.2.1]oct-3b-ylamine (**7**) (Dostert et al. 1984)

660g (5 mol) of 2,5-dimethoxytetrahydrofuran were heated at 80°C in 6 L of 0.1 N HCl for 1h. After cooling to 10°C, 803g (5.5mol)of acetone dicarboxylic acid and 460 ml of 12N HCl and 492g (6 mol) of sodium acetate were added with stirring. Then 589g (5.5 mol) of benzylamine were introduced and stirred overnight. Insoluble materials were filtered off and (5.5 mol) of hydroxylamine hydrochloride were added to the filtrate with stirring.

After 40 min., the reaction mixture was cooled and pH was adjusted to 7-8 with aqueous KOH under vigorous stirring. The precipitated oxime (**6**) was collected by filtration, washed with water and dried to give oxime (**6**) m.p. 124°C.

A solution of 2.1 mol of this oxime in 6L of amyl alcohol was heated to 80°C with stirring and 420g Na were carefully added to keep on refluxing. The mixture was poured into 3L of water cooled to 0°C. The aqueous layer was decanted, the organic layer was twice extracted with aqueous HCl. Basification with KOH and extraction with CH_2_Cl_2_ gave, after drying (Na_2_SO_4_) and evaporation compound **7**, b.p. 100-115°C (0.05mm). 1H NMR (CDCl_3_) 7.1-7.5 (m, 5, ArH), 3.5 (s, 2, CH_2_Ar), 3.15 (m, 2, H_1_ and H_5_), 2.6-3.25 (m, 1, J=40 Hz, axial H_3_), 1.1-2.2 (m, 10, NH_2_ and 4(CH_2_))

- 1. 8-phenylmethyl-8-azabicyclo[3.2.1]oct-3β-ylacetamide (**8**) and 8-azabicyclo[3.2.1]oct-3β-ylacetamide hydrochloride (**9**)(Dostert et al. 1984)

17 ml (0.302 mol) of acetyl chloride were added dropwise to a solution of 54.4 g (0.251 mol) of **7** and 41.7 ml (0.302 mol) of trimethylamine in 500 ml CH_2_Cl_2_ at 0°C with stirring for 12h. The mixture was poured into water at 0°C and extracted. The combined organic layers were dried (Na_2_SO_4_) and evaporated. Recrystallization from hexane-AcOEt (8:2) gave the acetamido derivative: m.p. 125°C. 80g of this intermediate (**8**) were stirred in 1000ml of EtOH and 1 ml of 5N etanolic-HCl, with 8g of Pd/C 10%, under pressure of hydrogen of 213 psi at 60°C for12h. Filtration and evaporation provided 40g of acetamido amine hydrochloride. Free base: m.p. 150°C

- 1. Synthesis of 8-methyl-8-azabicyclo[3.2.1]octan-3-one oxime (**14**)(Lewin et al. 1998),(Słowiński et al. 2011)

To the solution of 8-methyl-8-azabicyclo[3.2.1]octan-3-one (**13**) (0.05 mol) in ethanol (30 mL), a solution of hydroxylamine hydrochloride (6.9 g, 0.1 mol) in H_2_O (45 mL) was added. Subsequently, NaHCO_3_ (8.4 g, 0.1 mol) was added portionwise and the resulting mixture was heated under reflux for 30 min and stirred at room temperature for 24 h. Next, H_2_O (20 mL) was added and the mixture was extracted with CHCl_3_

(3 x 75 mL). The combined organic extracts were dried with magnesium sulphate, filtered, and the solvent was evaporated in vacuo to give crude **14** as an yellow oil.

1H NMR (CDCl_3_): 1.42-1.69 (m, 2H), 2.02 (br, 2H), 2.14 (d, 1H, J ) 14.9), 2.25 (dd, 1H, J ) 3.5, 15.6), 2.38 (s, 3H), 2.62 (dd, 1H, J ) 2.8, 15.0), 3.00 (d, 1H, J ) 15.4), 3.31 (br, 2H), 10.73 (br, 1H). 13C NMR(CDCl3): 155.01, 60.66, 59.92, 39.03, 36.99, 30.97, 27.20, 26.29.

- 1. Synthesis of 8-methyl-8-azabicyclo[3.2.1]oct-3b-ylamine (**15**) (Lewin et al. 1998),(Słowiński et al. 2011)

To a refluxing mixture of oxime **14** (0.032 mol) in absolute ethanol (120 mL), Na metal (7.0 g, 0.3 mol) was added portionwise followed refluxing for 4h. The solution was then cooled and H_2_O (150 mL) was carefully added. The mixture was acidified with concentrated aqueous HCl, the ethanol was evaporated in vacuo and the resulting solution was washed with diethyl ether (2x50mL).The aqueous phase was alkalised with a saturated aqueous solution of NaOH and extracted with diethyl ether (3 x 50 mL). The combined organic extracts were dried with magnesium sulphate, filtered, and the solvent was evaporated in vacuo to give crude **15** as an yellow oil. The oil was subjected to distillation.

1H NMR (CDCl3): 1.16 (br, 2H), 1.33-1.58 (m, 4H), 1.67-1.76 (m, 2H), 1.95-2.02 (m, 2H), 2.28 (s, 3H), 2.83-2.97 (m, 1H), 3.10-3.15 (m, 2H). 13C NMR (CDCl3): 60.45, 42.22, 41.64, 39.17, 26.06

- 1. (8-Methyl-8-azabicyclo[3.2.1]oct-3β-yl)-2-naphthamide (**16**) (Stefanowicz et al. 2016)

A solution of naphthalene-2-carboxylic acid (18.9g, 0.11 mol), ethyl chloroformate (10.5 mL, 0.11 mol) and triethylamine (16.5 mL, 0.12 mol) in anhydrous DMF (200 mL) was stirred for 30 min at 0ºC. After this time a solution of amine **15** (16.5g, 0.12 mol) in anhydrous DMF (40 mL) was added dropwise. The cooling bath was removed and stirring was continued for 24 h. The solvent was evaporated *in vacuo* and the residue was dissolved in CH_2_Cl_2_ (150 mL). The solution was washed with a 5% aqueous solution of Na_2_CO_3_ (3 x 50 mL), then with 5% NaOH (2 x 50 mL) and once with 100 mL of water. The organic layer was dried with magnesium sulphate, filtered, and concentrated *in vacuo*. The solid residue was purified by crystallisation from ethyl acetate. m. p. 200.7–201.8ºC;

**IR** (KBr) cm^-1^: ν3247 (NH), 1632 (CO); **ESI-HRMS** m/z calcd for C_19_H_22_N_2_OH (M + H)^+^ 295.1810, found: 295.1812.

**^1^H NMR** (500 MHz, CDCl_3_): d 8.22(C1’’H, ^4^J =1.0; m 7.87 (C4’’H, C5’’H, C8’’H); dd 7.77 (C3’’H) ^3^J= 8.5, ^4^J= 2.0; m 7.53 (C6’’H, C7’’H); d 6.06 (NH), ^3^J= 8.0; m 4.39 (C3H); pt 3.22 (C1H, C5H); s 2.31(C9H); m 2.08 (C2H(E),C4H(E)); m 1.98 (C6H(E),C7H(E)); m 1.77 (C6H(A),C7H(A)); td 1.66 (C2H(A),C4H(A)), ^2^J=^3^J_1_= 12.5, ^3^J_2_= 2.5.

- 1. N-(8-azabicyclo[3.2.1]oct-3β-yl)-2-naphthamide hydrochloride (1**7**) (Stefanowicz et al. 2016)

To a solution of 10.3g (0.035 mol) (8-methyl-8-azabicyclo[3.2.1]oct-3β-yl)-2-naphthamide (**16**) dissolved in a mixture of 150 mL DCM and 100 mL of 1,2-dichloroethane chilled to 0ºC and maintained at this temperature, 5.25g (4.0 mL, 0.037 mol) 1-chloroethyl chloroformate in 50 mL of 1,2-dichloroethane was added dropwise. The mixture was then refluxed for 2.5 h. Solvents were removed *in vacuo*, 100 mL of methanol was added to the residue and the mixture was refluxed for 90 min. After removing half of the methanol, the product precipitated. m.p. 160.2–163.4ºC; **IR** (KBr) cm^-1^: ν3389 (NH), 1653 (CO); **ESI-HRMS** m/z calcd for C_18_H_20_N_2_OH (M + H)^+^ 281.1654, found: 2815.1658.

**^1^H NMR** (500 MHz, CDCl_3_): d 8.02 (C1”H); m 7.85 (C5”H,C8”H); d 7.80 (C3”H,^3^J=8.5); m 7.57 (C4”H,C6”H,C7”H); m 4.28 (C3H); pt 4.13 (C1H,C5H); m 2.16 (C2H(E),C4H(E)); m 2.06 (C6H(E),C7H(E)); m 2.01 (C6H(A),C7H(A)); td 1.85 (C2H(A),C4H(A)),^2^J=^3^J_1_= 14.0,

^3^J_2_= 2.5.

- 1. References

Dostert P, Imbert T, Langlois M, et al (1984) Studies on the neuroleptic benzamides. III - Synthesis and antidopaminergic properties of new 3-nortropane derivatives. Eur J Med Chem - Chim Ther 19:105–110

Lewin AH, Sun G, Fudala L, et al (1998) Molecular Features Associated with Polyamine Modulation of NMDA Receptors. J Med Chem 41:988–995.

Słowiński T, Stefanowicz J, Dawidowski M, et al (2011) Synthesis and biological investigation of potential atypical antipsychotics with a tropane core. Part 1. Eur J Med Chem 46:4474–4488.

Stefanowicz J, Słowiński T, Wróbel MZ, et al (2016) Synthesis and biological investigation of new equatorial (β) stereoisomers of 3-aminotropane arylamides with atypical antipsychotic profile. Bioorganic Med Chem 24:3994–4007.

1. **UV spectra of the stereoisomers α and β, compounds 12u and 12m** **Figure 1** Superimposed UV spectra of the stereoisomers **12u** α and **12u** β

**Figure 2** Overlapping UV spectra of the stereoisomers **12m** α and **12m** β (disturbances in the spectrum of the α isomer are due to a very low concentration of this compound in the mixture).


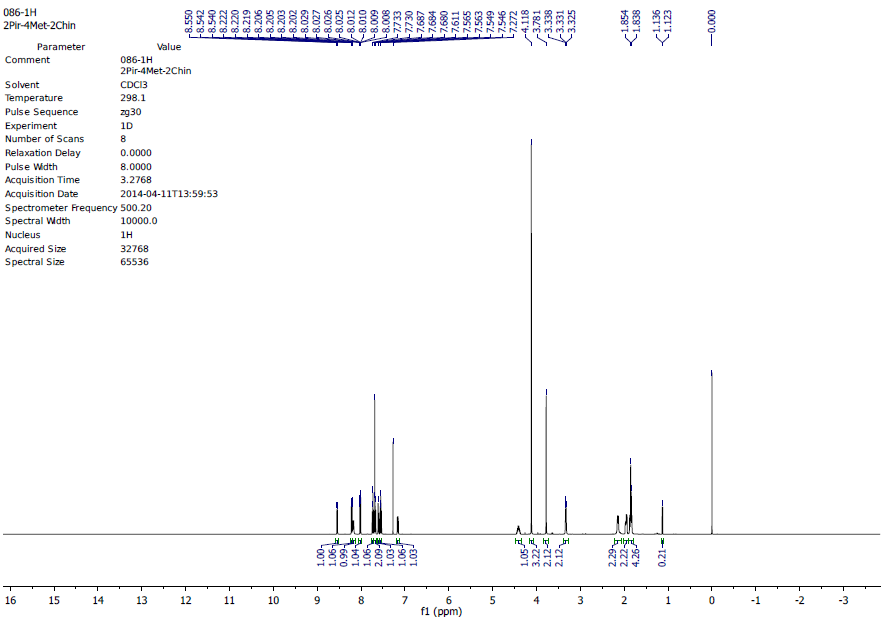

Supplement: Supplementary file 1 — Supplementary Information [file 44_2018_2203_MOESM1_ESM.doc]
